# Narrow-Bandgap Zero-Dimensional Chiral Hybrid Perovskites

**DOI:** 10.1021/jacs.6c07362

**Published:** 2026-07-24

**Authors:** Zhongyuan Li, Haitao Zhao, Jiasheng Xie, Shihai You, Shence Zhang, Gabriele Saleh, Liberato Manna

**Affiliations:** † Key Laboratory of Functional Molecular Solids, Ministry of Education, and College of Chemistry and Materials Science, 12514 Anhui Normal University, 241000 Wuhu, China; ‡ Research Institute of Frontier Science, 56711 Southwest Jiaotong University, Chengdu, Sichuan 610031, China; § Nanochemistry Line, 121451 Istituto Italiano di Tecnologia, Via Morego 30, 16163 Genova, Italy

## Abstract

Chiral hybrid perovskites (CHPs) are highly promising for next-generation chiroptoelectronics and spintronics. However, the incorporation of bulky organic chiral groups to impart chirality inevitably reduces structural dimensionality, leading to pronounced bandgap widening, which fundamentally limits their applications in narrow-bandgap optoelectronic devices. Here, for the first time, we report the construction of a pair of narrow-bandgap zero-dimensional (0D) CHPs, (*R*/*S*-MBA)_2_PtI_6_ (MBA = methylbenzylammonium), rationally designed by incorporating a 5d transition metal, Pt­(IV), with energetically accessible 5d orbitals, in a chiral hybrid structure. The materials exhibit a small band gap of 1.27 eV, among the lowest values reported to date for intrinsic chiral systems. (*R*/*S*-MBA)_2_PtI_6_ adopts a unique 0D structure with quasi-layered packing, in which the terminal ammonium groups of the chiral cations are uniformly embedded within the inorganic sublayer, forming an extended hydrogen-bonding network. Electronic structure calculations reveal that the I­(p) orbitals and their hybridization with Pt­(d) orbitals define the band-edge states, while hydrogen bonding further tunes the electronic energy levels through local octahedral distortion and lone pair stabilization. The intrinsic integration of such a narrow bandgap with chiroptical activity endows the materials with broadband photoresponse and high polarization selectivity (g = 0.26). Notably, it also shows sensitive X-ray response with a sensitivity of 1410.8 μC Gy^1–^ cm^–2^ and a low detection limit of 426.7 nGy s^–1^. These findings demonstrate that hydrogen-bonding-regulated 5d orbital-energy alignment provides an effective strategy to overcome the conventional trade-off between chirality-induced structural dimensionality reduction and bandgap widening in CHPs.

## Graphical abstract

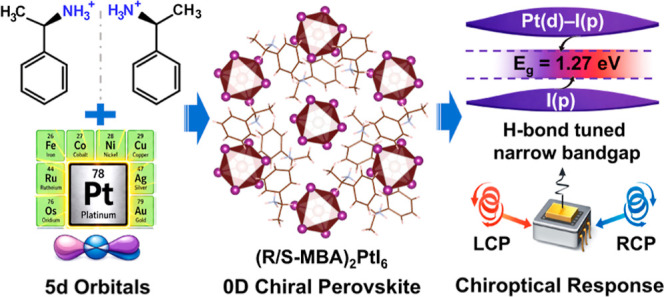

## ■Introduction

Chiral hybrid perovskites (CHPs), typically prepared by incorporating chiral organic cations into the lattice, have recently emerged as a distinctive class of semiconductor materials for optoelectronic and spintronic applications owing to their favorable electronic structures and intrinsic chiroptical activity.^1–3^ The incorporation of bulky chiral organic cations generally reduces the structural connectivity of the perovskite framework while simultaneously increasing the density of chiral components within the lattice, a configuration often associated with enhanced chiroptical responses in chiral perovskite systems.^4–6^ However, reduced structural connectivity often introduces pronounced quantum and dielectric confinement effects, leading to wider band gaps.^7,8^ Narrow-bandgap semiconductors are widely employed in photovoltaics,^9^ infrared detection,^10^ and thermoelectric energy conversion^11^ because of their ability to efficiently harvest low-energy photons and generate charge carriers. Therefore, achieving narrow-bandgap semiconductors in chiral perovskite systems represents a critical challenge yet remains highly desirable for advanced optoelectronic applications.

Narrow-bandgap behavior remains uncommon among reported CHPs and becomes increasingly difficult to achieve as structural dimensionality decreases. This limitation arises from the combined effects of reduced octahedral connectivity and the intrinsic electronic characteristics of commonly employed ns^2^ main-group metal centers. As dimensionality decreases, diminished structural connectivity weakens interoctahedral orbital coupling and suppresses band dispersion, thereby widening band gaps.^12–14^ This trend is evident in commonly chiral Pb-based halide systems, where 2D CHP such as (*R*/*S*-MBA)_2_PbI_4_ (MBA = methylbenzylammonium) typically exhibit band gaps exceeding 2.0 eV,^15^ and further reduction to 1D structures results in even wider band gaps.^16^ The situation is further aggravated when Pb^2+^ is replaced by heterovalent ns^2^ main-group cations such as Bi^3+^ or Sb^3+^, whose deeper-lying lone-pair states and weaker metal–halide s–p hybridization limit effective band-edge coupling and are inherently unfavorable for bandgap narrowing.^17^ Accordingly, the 1D bismuth (Bi)-based CHP (*R*-MBA)_2_Bi_2_I_8_ exhibits a band gap of ∼2.1 eV,^18^ while the representative 0D phase (*R*-MBA)_4_Bi_2_I_10_ has a gap near 2.5 eV.^19^ Reduced structural connectivity, together with the use of conventional ns^2^ main-group metal centers, fundamentally limits the realization of a narrow bandgap in CHPs.

To mitigate the bandgap widening commonly associated with reduced structural dimensionality, the introduction of transition-metal cations provides an alternative electronic-structure pathway. In particular, 5d transition metals provide energetically accessible d orbitals that can align with halogen p states and participate in frontier electronic levels.^20–22^ Such d-orbital involvement can lower the conduction-band edge relative to conventional ns^2^ main-group metal halide perovskites, thereby reducing the band gap without relying on increased band dispersion. Consistent with this rationale, previous studies on heavy transition-metal halides, including Pt- and Os-based vacancy-ordered all-inorganic 0D perovskites such as Cs_2_PtI_6_ and Cs_2_OsI_6_, have demonstrated relatively small band gaps through strong d–p hybridization,^23–25^ resulting in reduced band gaps compared with conventional ns^2^-based halide perovskites. However, these materials are typically achiral and structurally rigid, offering limited opportunities to tune their electronic structures through local structural interactions. In contrast, CHPs introduce additional structural degrees of freedom, particularly through asymmetric hydrogen bonding between chiral organic cations and inorganic frameworks, which can modulate local geometry and electronic energy levels.^26–28^ Despite this conceptual advantage, the realization of narrow-bandgap CHPs incorporating 5d transition-metal centers remains largely unexplored.

Here, we report a pair of narrow-bandgap 0D Pt­(IV)-based CHPs, (*R*/*S*-MBA)_2_PtI_6_, for the first time. The substantial coupling between Pt­(5d) and I­(p) orbitals, together with hydrogen-bonding-induced local lattice distortion and lone-pair stabilization, endow the materials with an exceptionally small bandgap of 1.27 eV, which is the smallest among the previously reported CHPs despite their 0D structural dimensionality. Benefiting from the narrow bandgap and intrinsic chiroptical activity, the materials exhibit broadband photoresponse spanning the UV-NIR region and enable sensitive detection of circularly polarized light with a large anisotropy factor of 0.26. In addition, we also demonstrate that the material has potential X-ray detection capabilities. These findings highlight a design strategy for achieving narrow-bandgap semiconductors in CHPs by coupling hydrogen-bond-mediated local structural modulation with 5d orbital-driven band-edge engineering.

## ■Results and Discussion

Enantiomeric cations *R*/*S*-MBA^+^ (Figure 1a, top left and 1b, top right), one pair of the most commonly used chiral inducers,^1^ were employed to construct CHPs. High-quality single crystals (SCs) of (*R*/*S*-MBA)_2_PtI_6_ were grown by slowly cooling a concentrated aqueous HI solution containing stoichiometric amounts of PtCl_4_ and *R*/*S*-MBA. Detailed synthetic procedures are provided in the Experimental Section. Single-crystal X-ray diffraction (SCXRD) revealed that (*R*-MBA)_2_PtI_6_ and (*S*-MBA)_2_PtI_6_ are antisymmetrically isostructural and crystallize in a chiral trigonal space group R3. The crystallographic parameters and refinement details are summarized in Table S1. In the chiral structure of (*R*/*S*-MBA)_2_PtI_6_, each Pt atom is octahedrally coordinated by six iodide ions, forming isolated [PtI_6_]^2–^ units separated by *R*/*S*-MBA^+^ cations, resulting in a typical 0D molecular structure. The Pt^4+^ ions in the crystals are precisely located on the 3-fold rotation axes; consequently, each [PtI_6_]^2–^ octahedron possesses C3 symmetry, featuring two distinct Pt–I bond lengths, four inequivalent cis I–Pt–I bond angles, and one type of trans I–Pt–I bond angle, as summarized in Tables S2–S7. The Pt–I bond lengths range from 2.66 to 2.69 Å, while the cis I–Pt–I bond angles vary from 88.3° to 91.5°, and the trans I–Pt–I bond angles are close to 180°. The SCs exhibit a triangular morphology at the millimeter scale when viewed along the *c*-axis, with the top surface corresponding to the polar (0001) plane (Figure S1). The experimentally measured powder X-ray diffraction (PXRD) patterns are in good agreement with those simulated from the SCXRD data, confirming the phase purity of the bulk (*R*/*S*-MBA)_2_PtI_6_ SCs (Figure S2). SEM images verify excellent crystal quality with a flat, smooth surface, and corresponding energy-dispersive spectroscopy (EDS) elemental mapping reveals a homogeneous distribution of Pt, I, C, and N throughout the crystals, consistent with the SCXRD results (Figure S3). X-ray photoelectron spectroscopy (XPS) was performed to determine the oxidation state of Pt. The characteristic peaks at binding energies of 311.10 and 314.59 eV, corresponding to the Pt 3d_5/2_ and 3d_3/2_ levels of Pt^4+^, respectively,^23^ confirm its +4 oxidation state (Figure S4). Thermogravimetric analysis indicates that both compounds exhibit similar thermal decomposition behavior and remain thermally stable up to approximately 175 °C (Figure S5). Furthermore, the compounds exhibit good environmental stability, with no detectable phase change after 2 months of storage under ambient conditions (Figure S6).

**Figure 1:**
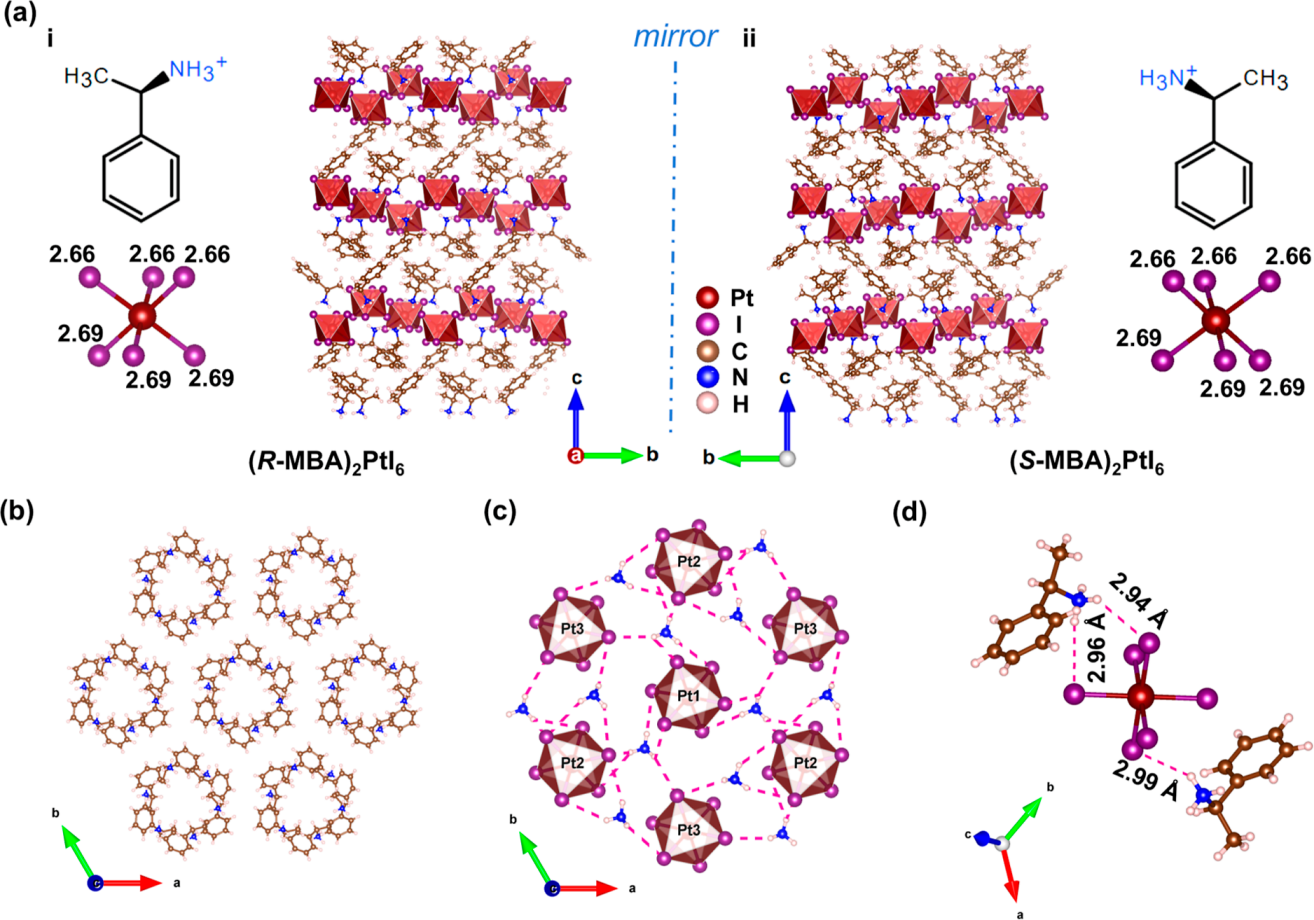
(a) Single-crystal structure of chiral (R/S-MBA)_2_PtI_6_, showing the chiral *R*/*S*-MBA^+^ cations and crystallographically isolated [PtI_6_]^2–^ octahedra. (b) Packing of the organic sublayer viewed along the *c* axis. (c) Packing of the inorganic sublayer within N–H···I hydrogen-bonding network (pink dashed line) viewed along the *c* axis. Only terminal ammonium ions from organic cations are displayed to present a hydrogen-bonding network. (d) N–H···I hydrogen-bonding surrounding [Pt(1)­I_6_]^2–^ octahedra. The other three equivalent hydrogen bonds are omitted.

Interestingly, the chiral structure exhibits a quasi-layered arrangement character. The inorganic [PtI_6_]^2–^ octahedra units are discretely arranged within the *ab* plane and stacked along the *c*-axis with a relative positional offset to form an inorganic sublayer, which is sandwiched between organic sublayers (Figure 1a). Meanwhile, the six phenyl groups of the organic cations arrange into a ring-like cavity that self-assembles into organic sublayers within the ab plane (Figure 1b), whereas the terminal ammonium groups of MBA^+^ penetrate into the inorganic sublayer, forming a hydrogen-bonding network with neighboring [PtI_6_]^2–^ ions (Figure 1c) and thereby stabilizing the lattice. This layered packing arrangement allows closer packing of the inorganic octahedra within the inorganic sublayer, yielding a shortened I···I distance of 4.05 Å between adjacent metal–halide octahedra (Figure S7). This distance is significantly shorter than that in typical fully isolated 0D hybrid metal halides (typically >5–6 Å).^29–31^ The closer interatomic spacing of the inorganic octahedral units is expected to facilitate electron transfer within the material upon photoabsorption. To gain a better understanding of the local structural difference in crystal symmetry induced by the chiral amine cations, the achiral analog (*rac*-MBA)_2_PtI_6_ SCs were also prepared for comparison. (*rac*-MBA)_2_PtI_6_ also adopts a 0D structure but crystallizes in the centrosymmetric trigonal R3̅ space group (Figure S8). The hydrogen bonding around [PtI_6_]^2–^ units is stronger in the chiral structure. Let us take the Pt(1) octahedron, [Pt(1)­I_6_]^2–^, as a representative example; the length of asymmetric NH···I hydrogen bonds (HBs) in (*R*-MBA)_2_PtI_6_ ranges from 2.94 to 2.99 Å (Figure 1d), in contrast to the larger NH···I HB length in (*rac*-MBA)_2_PtI_6_ (3.00–3.07 Å, Figure S9). Hirshfeld surface analysis was adopted to gain quantitative insights into the crystal packing of (MBA)_2_PtI_6_ (Figure S10–S12). A large fraction of the intermolecular contacts is H···H (∼50%), with C···H and H···C accounting for 13% and 16% in chiral and achiral compounds, respectively. No C···C contacts are detected in either (*R*/*S*-MBA)_2_PtI_6_ or (*rac*-MBA)_2_PtI_6_. This Hirshfeld contact scenario indicates that molecules are mostly held together by nondirectional dispersion forces, with some contribution from C–H···π interactions and no detectable π···π stacking. The molecules-PtI_6_ contacts are entirely of HB type. They play a more prominent role in (*R*/*S*-MBA)_2_PtI_6,_ where they account for ∼34% of the total intermolecular interactions, compared to ∼30% in the achiral (*rac*-MBA)_2_PtI_6_ structure. The distortion of metal-halide octahedral units is closely correlated with HB interactions that influence chirality transfer in chiral hybrid perovskites.^6^ We calculated the distortion of [PtI_6_] octahedra by employing widely adopted quantities, the bond length deviation (λ_oct_) and bond angle variance (σ^2^), defined as:^32,33^

1 $$\lambda_{oct}=\frac{1}{6}\sum_{i=1}^{6}{\left(\frac{d_{n}-d_{0}}{d_{0}}\right)}^{2}$$

2 $$\sigma^{2}=\frac{1}{11}\sum_{i=1}^{12}{\left(\theta_{i}-90^{{}^{\circ}}\right)}^{2}$$

where d_0_ is the average Pt–I bond length, d_n_ refers to the *n*-th of the six individual Pt−I bond lengths, and θ_i_ is the *i*-th cis I–Pt–I bond angle of the octahedra. Both distortion indicators (Table S8) are greater for (*R*/*S*-MBA)_2_PtI_6_ than for (*rac*-MBA)_2_PtI_6_. The strong HB interactions are more likely to induce symmetry-breaking distortions in the inorganic sublattice, thereby facilitating the transfer of molecular chirality to the extended perovskite framework. Moreover, the distortion of the metal-halide octahedral units is expected to directly influence the material’s electronic properties, as discussed below.

UV–Vis–NIR absorption spectroscopy was employed to investigate the optical properties of (*R*/*S*-MBA)_2_PtI_6_ (Figure 2a). Both compounds exhibit nearly identical ultrabroad absorption bands extending into the NIR region, with absorption edges at 975 nm, corresponding to optical band gaps of approximately 1.27 eV. To the best of our knowledge, the bandgap value is smaller than that of most reported intrinsic 3D, 2D, and 1D perovskite analogs, with the exception of the toxic Tl-based perovskite Cs_2_AgTlBr_6_ (0.95 eV) and unstable Sn­(II)-based hybrid semiconductor EtVSn_2_I_6_ (1.10 eV) (Figure 2b and Table S9). DFT simulations^34–36^ were performed to study the electronic structure of (MBA)_2_PtI_6_ and to rationalize its small band gap. The band edges exhibit a low dispersion, implying a strong localization of electronic states within PtI_6_ octahedra. While the band gap is indirect, the low dispersion makes the direct band gap easily accessible, as it lies only ∼3 meV above the indirect gap (Figure S13). The density of states (DOSs) and partial charge density (Figures 2c-d and S14) indicate that the valence and conduction bands are formed by I­(p) lone pairs and Pt­(*d*)-I­(p) antibonding orbitals, respectively. The small band gap on this and related materials, compared to that of p-block metal halides, can be traced back to the role of Pt 5d orbitals. These, indeed, have an energy that is sizably lower than the p orbitals of elements like Pb, Sn, or Bi, and slightly lower than the I­(p) orbitals.^37^ Therefore, even though 5d orbitals efficiently hybridize with I­(p) orbitals, the empty Pt­(*d*)-I­(p) antibonding states lie only 1–1.5 eV above the I­(p) orbitals forming the valence band, hence the small band gap. In fact, the valence band maximum (VBM) and conduction band minimum (CBM) are unevenly distributed among the various PtI_6_ octahedra. The presence of five symmetry-independent octahedra, three in (*R*/*S*-MBA)_2_PtI_6_ and two in (*rac*-MBA)_2_PtI_6_, makes it possible to study how their geometry affects the energy of VBM and CBM, hence the band gap. As shown in Figures 2d and S15, the VMB is invariably localized on the I atoms that are not involved in HBs. Indeed, HBs are known to stabilize the acceptor lone pairs, thereby lowering their energy.^38^ The conduction band Pt–I antibonding states are generally spread through the whole PtI_6_ octahedron. The CBM, however, is localized on those PtI_6_ octahedra that feature the greatest distortion σ^2^ value (Table S8), likely as a result of nonoptimal Pt­(*d*)-I­(p) overlap and consequent lowering of the bonding–antibonding splitting. Overall, the effect of HBs is to lower both the VBM and the CBM via lone-pair stabilization and octahedral distortion, respectively. Their effect on the band gap depends on which of these two mechanisms dominates. In (MBA)_2_PtI_6_, we observe stronger HBs and a lower band gap (*R*/*S*-MBA)_2_PtI_6_ than in (*rac*-MBA)_2_PtI_6_ Figures 2a and S17), indicating that the distortion effect dominates over lone-pair stabilization.

**Figure 2:**
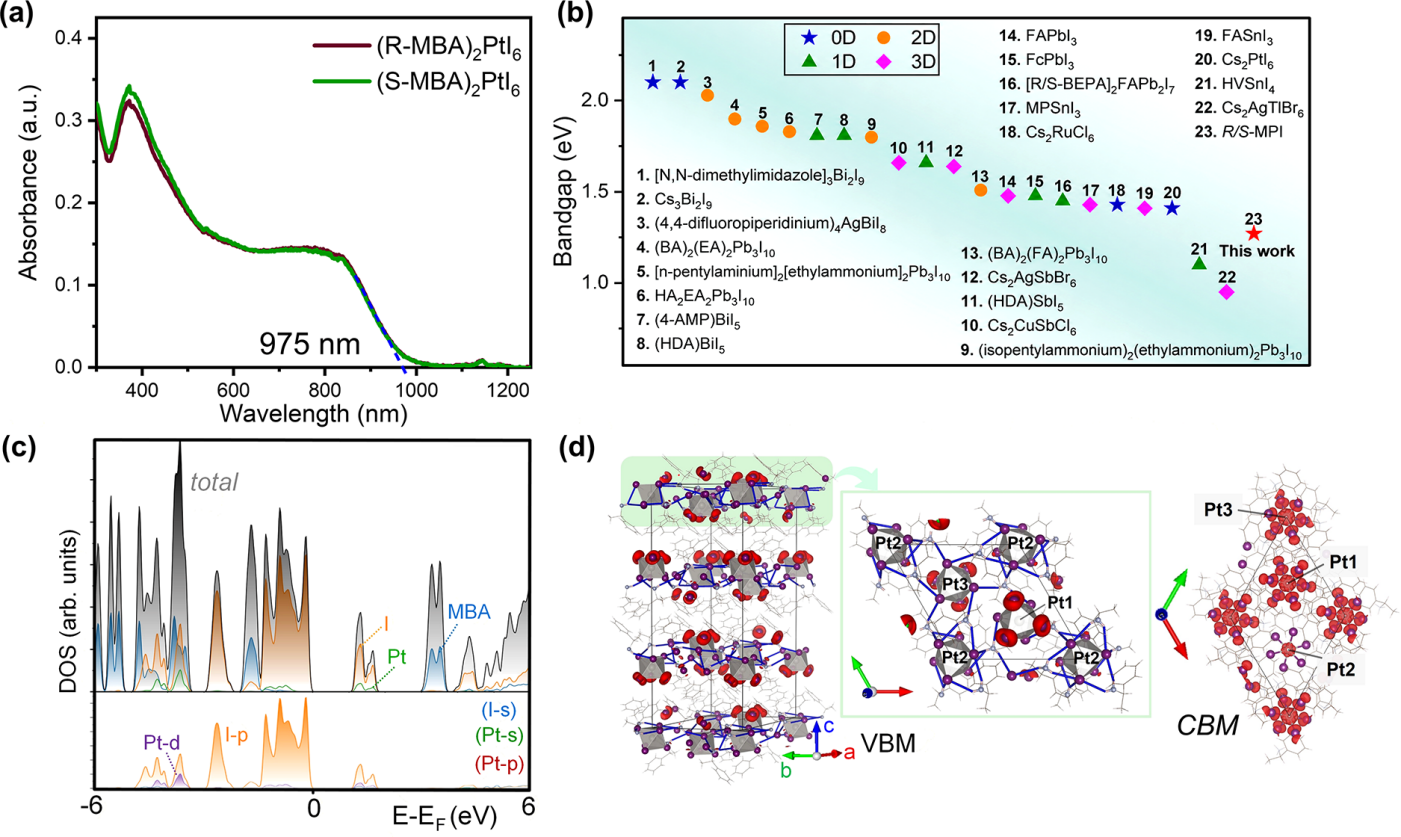
(a) UV–Vis–NIR absorption spectra of (*R*/*S*-MBA)_2_PtI_6_ single crystals, showing an ultrabroad absorption extending to ∼975 nm, corresponding to an optical band gap of ∼1.27 eV. (b) Comparison of bandgap values for representative 3D, 2D, 1D, and 0D halide perovskites (see Table S9 for detailed data and references). Density of states (DOS, c) and electron density distribution (d) of the valence band maxima (VBM) and conduction band minima (CBM) for (*R*-MBA)_2_PtI_6_. The DOS projections onto atoms (top) and atomic orbitals (bottom) are shown, obtained using the PBE functional. The electron density distribution is shown as red isosurfaces, with energy ranges relative to the Fermi level: VMB = −0.4/0.0 eV, CBM = 0.5/1.3 eV. N-H···I hydrogen bonds are shown in the VBM as blue lines. The labelling of Pt atoms is the same as in Tables S2-S8, with Pt2 forming the least distorted octahedron (lowest σ2).

The narrow bandgap indicates promising broadband photoactivity spanning the UV-NIR regions. Accordingly, a two-terminal lateral photodetector (PD) with the configuration Ag/(*R*-MBA)_2_PtI_6_/Ag was fabricated using high-quality (*R*-MBA)_2_PtI_6_ SCs to evaluate its photoresponse performance (Figure S18). As shown in Figure 3a, (*R*-MBA)_2_PtI_6_ exhibits pronounced photoresponses across a wide wavelength range from UV (405 nm) to NIR (785 nm), consistent with its narrow bandgap. Under identical illumination density, the maximum photocurrent response was observed at 520 nm (Figure 3a, inset); therefore, this wavelength was chosen as the representative light for further evaluation of photodetection performance. Figure S19 shows the current–voltage (I–V) traces of the PD device measured in the dark and under light illumination. The symmetric and linear behavior indicates good Ohmic contact and low contact resistance at the electrodes, enabling efficient charge collection. Figure 3b presents the time-dependent current density (I–t) curves of the PD device under different light power densities (1.49–104.14.1mw cm^–2^). As shown, the dark current remains highly stable, and the photocurrent density increases markedly with increasing light intensity, demonstrating a reliable and excellent photoresponse. The PD device exhibits a dark current of 1.84 nA at 3 V, which increases to 32.04 nA under a light intensity of 104.14 mW cm^–2^, producing a photocurrent of 30.20 nA. Accordingly, the on/off ratio was calculated to be 16.4, indicating that (*R*/*S*-MBA)_2_PtI_6_ is an efficient semiconductor material for photodetection. The power-dependent photocurrent follows a relationship of I_ph_ ∝ P^n^ (Figure S20), where the argument of the exponential (n = 0.47) is lower than 1, suggesting partial recombination losses of photogenerated carriers, likely arising from the presence of trap states within the material.^39,40^ Correspondingly, the responsivity (R) and the detectivity (D*) were calculated to be 90.1 μA W^1–^ and 1.31 × 10^9^ Jones, respectively (Figure S21). The temporal photoresponse of the device was characterized by a rise time (t_r_) of 0.66 ms and a fall time (t_f_) of 0.79 ms (Figure 3c), where t_r_ and t_f_ are defined as the time required for the photocurrent to increase from 10% to 90% and decrease from 90% to 10% of its maximum value, respectively. The relatively prolonged fall time can also be attributed to carrier trapping and slow release processes.

**Figure 3:**
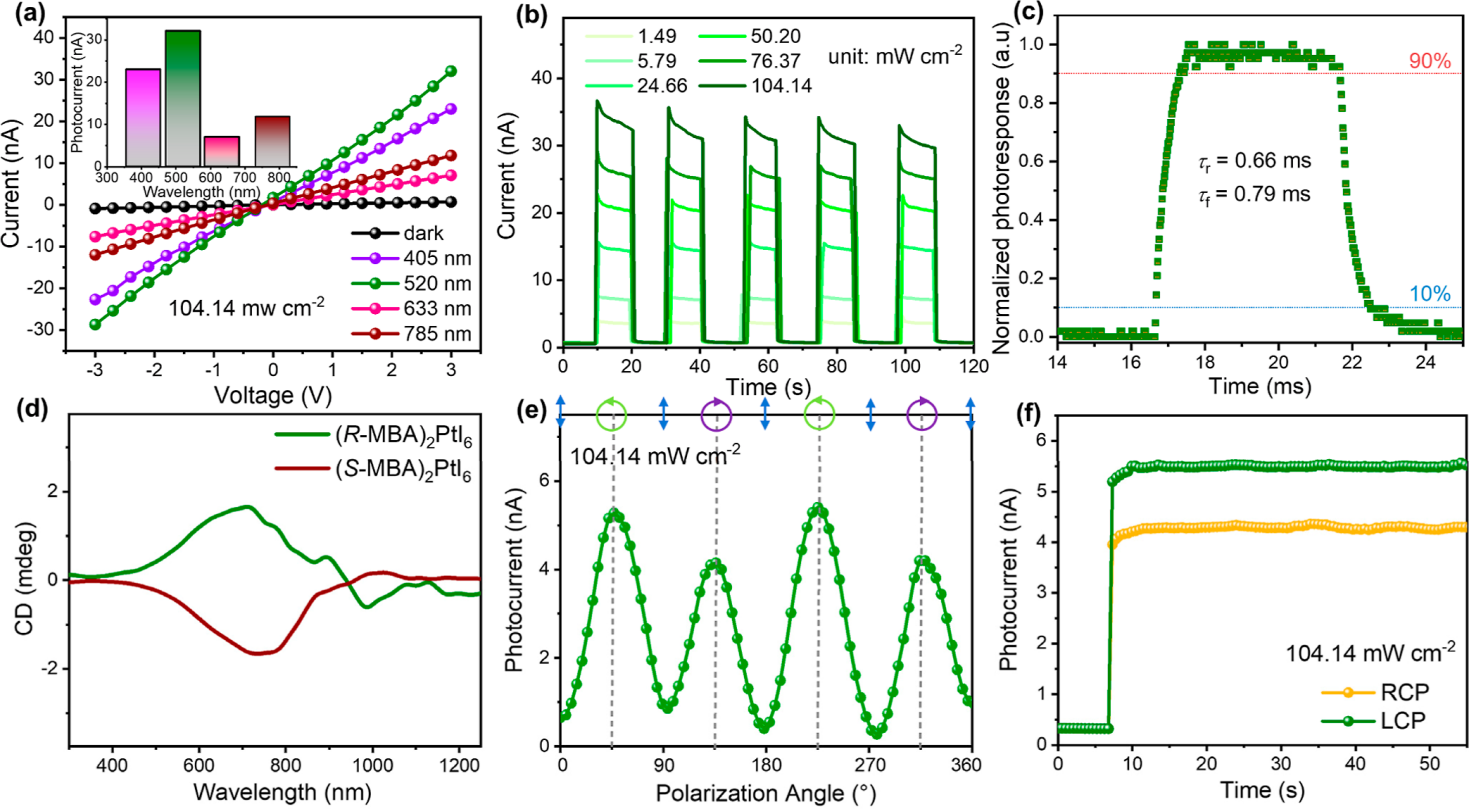
(a) I–V characteristics of the (*R*-MBA)_2_PtI_6_ photodetector measured in the dark and under illumination at different wavelengths (405, 520, 633, and 785 nm). (b) I–t curves under 520 nm illumination at varying light power densities. (c) Temporal photoresponse showing the rise and decay times of the (*R*-MBA)_2_PtI_6_ device. (d) Solid-state CD spectra of (*R*/*S*-MBA)_2_PtI_6_. (e) Short-circuit photocurrent of the (*R*-MBA)_2_PtI_6_ PD as a function of the rotation angle of the quarter-wave (λ/4) plate under constant illumination intensity. (f) Time-dependent photocurrent under LCP and RCP light at 520 nm with identical power density.

Given the chiral structure, the chiral optical properties of (*R*-MBA)_2_PtI_6_ were further studied. As shown in Figure 3d, (*R*-MBA)_2_PtI_6_ exhibits clear circular dichroism (CD) signals that are opposite in sign to those of its enantiomer (*S*-MBA)_2_PtI_6_ at nearly identical wavelengths. Notably, the distinct CD signals are located within the broadband absorption range from 400 to 950 nm, consistent with the absorption spectra in Figure 2a, most likely from the inorganic component in the chiral structure. The CD spectra of (*R*/*S*-MBA)_2_PtI_6_ are clearly distinct from those of the chiral inducers R/S-MBA, with apparent CD signals in the short-wavelength region (<350 nm) attributable to the sole chiral organic molecules (Figure S22). This result indicates the direct transfer of chirality from organic cations to the perovskite network. This is in line with previous works on various chiral hybrid perovskites.^41–43^ Coupling of photocurrent response and chiroptical activities should lead to circularly polarized light (CPL) detection capability, and this was investigated on (*R*-MBA)_2_PtI_6_. Because the optimal photocurrent response was obtained at 520 nm, this wavelength was still used as the representative light for CPL detection. The polarization state of CPL was controlled using a linear polarizer and a rotatable quarter-wave plate. The laser beam was first linearly polarized by the polarizer and then converted into circularly polarized light by the quarter-wave plate. By setting the fast axis of the quarter-wave plate at 45° and 135° relative to the incident polarization direction, left-handed circularly polarized (LCP) and right-handed circularly polarized (RCP) light were generated, respectively (Figure S23). As shown in Figure 3e, the photocurrent under LCP light illumination was higher than that under RCP light illumination with the same intensity. Across multiple switching cycles and extended exposure times, the photocurrent generated by RCP and LCP illumination did not significantly attenuate or approach zero, indicating reproducible and stable CPL detection (Figure 3f). Accordingly, the anisotropy factor for photocurrent, g_Iph_, was deduced according to g_lph_ = 2­(I_L_-I_R_)/(I_L_ + I_R_), in which I_L_ and I_R_ represent the photocurrent under LCP and RCP illumination.^44^ The g_lph_ value was 0.26 for (*R*-MBA)_2_PtI_6_, the highest among the several reported cases of 0D lead-free chiral perovskites, and also comparable to most of the 2D and 1D lead-based chiral perovskites (Table S10). The relatively large g_lph_ value is likely associated with the synergistic effects of strong Pt-induced spin–orbit coupling, chirality-transfer-mediated distortion of the inorganic framework, and the unique quasi-layered packing of the 0D structure. Specifically, the anisotropic environment produced by asymmetric HBs, combined with the strong spin-orbit interaction of Pt, can give rise to Rashba-like effects that lead to spin selectivity.^26,45,46^ In addition, the short interoctahedral I···I contacts and extended hydrogen-bonding network facilitate carrier transport between neighboring octahedra.^31^ As a result, spin-dependent carrier generation, transport, and collection processes can amplify the photocurrent contrast beyond that expected solely from the optical CD response. Additional CPL photodetection measurements at 785 nm (Figure S24) further confirm the persistence of chiral photoresponse in the low-energy absorption region. Although the g_lph_ decreases to 0.11, likely due to weaker absorption and reduced photocarrier generation near the band edge, distinguishable responses to LCP and RCP light can still be observed. These results reveal that Pt­(IV)-based 0D chiral perovskites are promising for highly anisotropic detection of circularly polarized light.

Considering its appealing photocurrent response and incorporation of heavy Pt and I elements, (*R*-MBA)_2_PtI_6_ is also expected to be highly promising for X-ray detection. Before evaluating the X-ray detection performance, the dark-current characteristics of (R-MBA)_2_PtI_6_ were investigated. Despite its narrow bandgap of 1.27 eV, the SC exhibits a high bulk resistivity of 4.81 × 10^9^ Ω cm (Figure S25), which remains substantially higher than those of typical 3D MAPbX_3_ (X = Cl, Br, I) SCs (10 ^7^∼10^8^ Ω cm).^47,48^ In addition, the activation energy for ion migration was determined to be 0.48 eV (Figure S26), exceeding that reported for typical narrow-bandgap perovskite MAPbI_3_ (0.26 eV).^49^ As a result, a low dark-current noise level of ∼10^–13^ A Hz^–1/2^ was observed in the frequency range of 1–100 Hz (Figure S27), indicating effective suppression of leakage current and ionic transport. These characteristics are beneficial for achieving stable and sensitive X-ray detection. Accordingly, the absorption coefficient of (*R*-MBA)_2_PtI_6_ was first evaluated using the NIST XCOM photon cross-section database,^50^ and several representative semiconductor materials commonly used for X-ray detection, including Si, α-Se, CdTe, and MA_3_Bi_2_I_9_, were also simulated over a broad photon energy range from 1 to 400 keV for comparison. As shown in Figure 4a, the linear absorption coefficient of (*R*-MBA)_2_PtI_6_ is significantly higher than that of Si across the entire energy spectrum and exceeds that of commercial α-Se in the photon energy range of 33–400 keV. Moreover, it is comparable to that of the high-performance perovskite X-ray detection material MA_3_Bi_2_I_9_, indicating excellent X-ray attenuation. Figure S28 presents the attenuation efficiency of these materials for 50 keV hard X-ray photons, corresponding to the maximum photon energy of the X-ray tube used in this work. In general, a higher attenuation efficiency enables more complete absorption of incident X-ray photons using thinner detector layers. Specifically, at a thickness of 1 mm, (*R*-MBA)_2_PtI_6_ can attenuate approximately 93% of the incident X-ray photons, which is comparable to that of the state-of-the-art perovskite MA_3_Bi_2_I_9_ (92%) and substantially higher than that of Si (9.71%). Under X-ray radiation, the X-ray detector based on (*R*-MBA)_2_PtI_6_ exhibited a mobility-lifetime product (μτ) up to 2.38 × 10^–5^ cm^2^ V^–1^ (Figure 4b), which is much larger than those of commercial α-Se films (≈10^–7^ cm^2^ V^–1^) and MAPbI_3_ polycrystalline films (2 × 10^–7^ cm^2^ V^–1^),^51^ and comparable to those of several lead-free 0D perovskite SCs, such as Cs_3_Bi_2_I_9_ (2.03 × 10^–5^ cm^2^ V^–1^),^52^ FA_3_Bi_2_I_9_ (2.4 × 10^–5^ cm^2^ V^–1^),^53^ and (S-BPEA)_4_Bi_2_I_10_ (1.4 × 10^–5^ cm^2^ V^–1^).^54^ Figures 4c and S29 show that the photocurrent density increases markedly with increasing X-ray dose rate from 33.7 to 169.1 μGy s^–1^ under all applied bias voltages (10–50 V), demonstrating a reliable and excellent X-ray response. Notably, even under a relatively high bias of 50 V, the dark current remains highly stable, in sharp contrast to the pronounced dark-current drift commonly observed in traditional 3D perovskite detectors, highlighting the excellent operational stability for direct X-ray detection. The photocurrent density increases linearly with the X-ray dose rate under a given bias voltage (Figure 4d). The sensitivity, extracted from the slope of the fitted curves, is determined to be 269.2 μC Gy^1–^ cm^–2^ at 10 V and increases to 1410.8 μC Gy^1–^ cm^–2^ at 50 V (Figure S30), which is higher than that reported for Bi­(III)-based 0D perovskite materials for X-ray detection, such as FA_3_BiBr_6_ (231 μC Gy_air_^–1^ cm^–2^ @ 100 V mm^–1^) (231 μC Gy_air_^–1^ cm^–2^@100 V mm^–1^),^55^ FA_3_Bi_2_I_9_ (598.1 μC Gy_air_^–1^ cm^–2^ @ 500 V),^53^ (HIS)­BiI_5_ (1000 μC Gy_air_^–1^ cm^–2^ @ 50 V),^56^ and (C_9_H_14_N)_3_Bi_2_I_9_ (1373.27 μC Gy_air_^–1^ cm^–2^ @ 5.71 V mm^–1^).^57^ The detection limit was further evaluated according to the International Union of Pure and Applied Chemistry (IUPAC) definition, which specifies the minimum detectable dose rate at a signal-to-noise ratio (SNR) of 3. Figure 4e shows the SNR as a function of X-ray dose rate at a bias voltage of 30 V. By fitting the SNR–dose rate relationship, a low detection limit of 426.7 nGy s^–1^ was obtained, which is 13 times lower than that required for routine medical diagnostics (5.5 μGy s^–1^),^58^ indicating the potential for reliable low-dose X-ray detection and reduced radiation exposure risks. Finally, the operational stability of the fabricated X-ray detector was evaluated. As shown in Figure 4f, the dark current was continuously monitored under a high bias voltage of 50 V, from which the baseline drift (I_drift_) of the (*R*-MBA)_2_PtI_6_ detector was calculated to be 3.46 × 10^–7^ nA cm^–1^ s^–1^ V^–1^. This value is approximately 4 orders of magnitude lower than that of conventional 3D perovskite detectors (∼10^–3^ nA cm^–1^ s^–1^ V^–1^),^59^ and is also lower than many recently reported low-dimensional lead-free perovskite detectors, i.e., (PPA)_2_BiI_5_ (1.0 × 10^–4^ nA cm^–1^ s^–1^ V^–1^),^60^ (1,3-BMACH)­BiI_5_ (9.6 × 10^–6^ nA cm^–1^ s^–1^ V^–1^; 1,3-BMACH = 1,3-bis­(aminomethyl)­cyclohexane),^61^ and (4-AMP)­BiI_5_ (1.51 × 10^–5^ nA cm^–1^ s^–1^ V^–1^; 4-AMP = 4-(aminomethyl)­pyridine).^62^ In addition, the operational stability of the device was further evaluated under continuous X-ray irradiation. As shown in Figure S31, the device can still maintain a stable photocurrent response throughout the measurement under continuous X-ray irradiation at a high dose rate (566.7 μGy_air_ s^–1^) and bias voltage (50 V), exhibiting only a slight degradation of approximately 10% after nine consecutive on/off irradiation cycles over an extended testing period. The good operational stability can be attributed to the suppressed ion migration in the unique 0D crystal structure. Consequently, Pt-based 0D perovskite (*R*-MBA)_2_PtI_6_ shows sensitive and stable X-ray detection performance, facilitating its application in photoelectronic fields.

**Figure 4:**
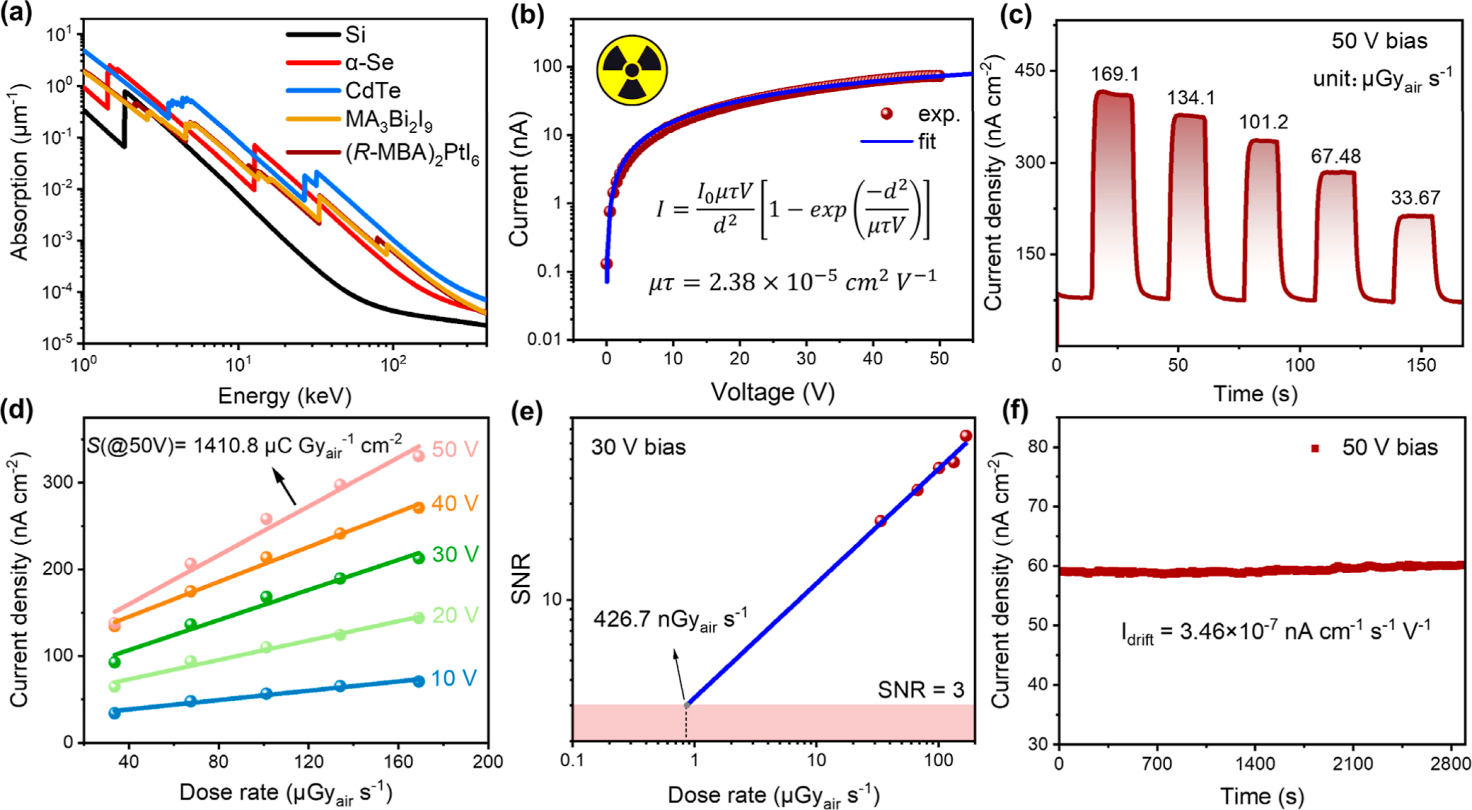
(a) Calculated X-ray absorption coefficients of (*R*-MBA)_2_PtI_6_ compared with representative detector materials (Si, α-Se, CdTe, and MA_3_Bi_2_I_9_). (b) Bias-dependent photocurrent under X-ray irradiation, yielding a mobility–lifetime (μτ) product of 2.38 × 10^–5^ cm^2^ V^–1^. (c) J–t curves recorded under X-ray irradiation at different dose rates at 50 V. (d) X-ray-induced current density as a function of dose rate at various applied biases (10–50 V). (e) SNR versus dose rate at 30 V, giving a detection limit of 426.7 nGy s^–1^. (f) Dark current drift under 50 V bias for (*R*-MBA)_2_PtI_6_.

## ■Conclusions

In summary, we have reported a pair of narrow-bandgap 0D CHPs, (*R*/*S*-MBA)_2_PtI_6_, enabled by incorporating the 5d transition metal Pt­(IV) in a chiral hybrid framework. The materials exhibit intrinsic chirality together with an exceptionally small band gap of 1.27 eV, among the lowest reported for CHPs. The band gap narrowing is governed by Pt­(5d)–I­(p) orbitals hybridization, further tuned by hydrogen-bonding-induced lattice distortion and lone-pair stabilization, as demonstrated by electronic structure analysis. Consequently, (*R*/*S*-MBA)_2_PtI_6_ shows broadband photoresponse spanning the UV–NIR region, high circular polarization selectivity (*g* = 0.26), and excellent X-ray detection performance. These findings reveal that 5d-driven orbital hybridization with hydrogen-bond-mediated local structural regulation provides an effective strategy to overcome the conventional trade-off between reduced dimensionality and bandgap widening in CHPs. This work offers a new direction for developing narrow-bandgap chiral semiconductor materials for novel spintronic and related applications.

## Supplementary Material



## Notes

Experimental procedures; single-crystal photograph; powder X-ray diffraction; SEM and EDS element mappings; XPS spectra; TG curves; single-crystal structures of achiral structure; Hirshfeld surface analysis; calculated band structure; partial density of states; calculated electron density distribution; calculated density of states; UV–visible–NIR absorption spectra; characteristic of photodetector performance; calculation of polarization value; CD spectra; characteristic of X-ray detectionperformance; crystal data and structure refinement parameters; calculation of distortion; bandgap comparison; performance comparison of CPL detector (PDF)
