# Supplementary material for: Narrow-Bandgap Zero-Dimensional Chiral Hybrid Perovskites
Source: J Am Chem Soc. 2026 Jul 24;148(30):32167–76. doi: 10.1021/jacs.6c07362 (PMC13450394; doi:10.1021/jacs.6c07362)
Supplement: Supplementary file 1 [file ja6c07362_si_001.pdf]

Supporting Information

Narrow-Bandgap Zero-dimensional Chiral Hybrid Perovskites

Zhongyuan Li,[1,3] Haitao Zhao,[1] Jiasheng Xie,[2] Shihai You,[2*] Shence Zhang,[3] Gabriele Saleh,[3*] and Liberato Manna[3*]

[1] Key Laboratory of Functional Molecular Solids, Ministry of Education, and College of Chemistry and Materials Science, Anhui Normal University, 241000 Wuhu, China

[2] Research Institute of Frontier Science, Southwest Jiaotong University, Chengdu, Sichuan 610031, China

[3] Nanochemistry Line, Istituto Italiano di Tecnologia, Via Morego 30, 16163 Genova, Italy

[*]Email: shihai.you@swjtu.edu.cn (S.Y.); g*abriele.saleh@iit.it (G.S.); liberato.manna@iit.it (L.M.)*

Experimental Section

Materials and Chemicals. All chemicals, platinum chloride ($PtCl_4$, 99.9%, Aladdin), hydroiodic acid solution (HI, 48% in solution, Aladdin), (*R*)-(+)-α-Methylbenzylamine (*R-MBA*, 98%, Aladdin), (*S*)-(-)-α-Methylbenzylamine (*S*-MBA, 98%, Aladdin), for the synthesis were bought and used without further purification.

Single Crystal Growth: High-quality single crystals were grown by slowly cooling a concentrated aqueous solution of HI containing stoichiometric amounts of $PtCl_4$ and *R/S*-MBA. First, 0.1 mmol of $PtCl_4$ was added to a 2 mL HI solution, which was then heated and stirred at 120 °C for 0.5 h until the solution became clear. The temperature was then lowered to 60 °C. Meanwhile, 0.5 mmol (65 μl) of *R/S*-MBA was added to another 0.5 ml HI solution and stirred in an ice-water bath for 0.5 h to protonate the amines. Subsequently, the protonated *R/S*-MBA solution was transferred into the Pt-HI solution, and the temperature was maintained at 60 °C for an additional 0.5 h. Finally, the solution was transferred to a 60 °C oven immediately and cooled at 2.5 °C day^{-1}. Then, the bulk single crystals of (R/S-MBA)$_2$PtI$_6$ were precipitated during slow cooling.

Single-Crystal and Powder X-ray Diffraction: Single-crystal X-ray diffraction (SCXRD) was performed on (R/S-MBA)$_2$PtI$_6$ and (*rac*-MBA)$_2$PtI$_6$ using a Oxford Gemini S Ultra system with Mo Kα radiation (λ = 0.77 Å). The multi-scan absorption corrections were applied using the CrysAlis program. The crystal structure was solved and refined by least squares on F2 using the SHELXTL and $OLEX^2$ programs. The VESTA software was used to depict the crystal structure. The simulated XRD pattern was calculated using SCXRD and extracted using VESTA. The PXRD patterns were collected using a Bruker D8 ADVANCE with Cu Kα (λ = 1.5406 Å) operating at 40 kV/40 mA.

Scanning Electron Microscopy (SEM) and Energy-Dispersive X-ray Spectroscopy (EDS): Scanning electron microscopy (SEM) images, together with elemental mapping, were obtained using a field-emission scanning electron microscope (Hitachi 8100).

X-ray Photoelectron Spectroscopy (XPS): The XPS measurements were performed using a PHI Quantum 2000 instrument (Ulvac-Phi, Inc., Tokyo, Japan).

Thermogravimetric (TG) Analysis: The TGA measurements were performed using a thermal analyzer (NETZSCH TG 209 F1). The samples were heated from 25 to 800 °C at a rate of 10 °C min^{-1} under a N_2 flow rate of 40 mL min^{-1}.

Absorption Spectrum: The optical absorption measurements were carried out using a Shimadzu UV-2600I UV– vis–NIR spectrometer operating in the 200−1200 nm region at room temperature.

Solid-State Circular Dichroism (CD) Spectrum: A JASCO-1500 CD spectrometer is used for the solid-state CD measurements. KBr pellets are used as the matrices, the scanning rate is 200 nm min-1, and the data interval is 0.5 nm. The chiral compounds were mixed with KBr by weight and fully ground to obtain powder samples, then pressed into uniform, transparent pellets for CD tests.

Device Fabrication: The two-terminal single-crystal device, Ag/(R-MBA)$_2$PtI$_6$ SC/Ag, was fabricated by coating conductive silver paste (SPI, US) on the two ends of an (R-MBA)$_2$PtI$_6$ single crystal along the c-axis. These crystals were fished out from the solution and dried in a vacuum oven at 50 °C. Thereafter, they were used for device fabrication without further surface treatment. A typical size of a used crystal is about 1 (width) × 1 (thickness) × 1 (length) mm^3 for X-ray detection. Thus, the active area for current-density

calculation is 0.01 cm^2, and the electrode spacing is 1 mm.

Ultraviolet-Visible Light Detection: The current-voltage (I-V) traces and current-time (I-t) curves were recorded using a high-precision electrometer (Keithley 6517B, USA). Multiple-wavelength fiber-pigtailed laser diodes, including 405, 520, 633, and 785 nm (Thorlabs, USA), were used as light sources. The incident light intensity was measured by a PM100D optical power meter (Thorlabs, USA).

Calculations of Responsivity (R) and Detectivity (D*)[1]: R and D* can be calculated by the following equation, respectively,

$$R = \frac{I_{ph}}{P_i S} = \frac{I_{light} - I_{dark}}{P_i S} \tag{1}$$

$$D^* = \frac{R\sqrt{S}}{\sqrt{2eI_{dark}}} \tag{2}$$

where I_{ph} is photocurrent, I_{dark} and I_{light} represent the currents measured in the dark and under light illumination, respectively. P_i is the incident light intensity, S is the effective illumination area, and e is the unit charge. Assuming that the noise current is mainly contributed by the shot noise from dark current.

X-ray Detection: A commercially available X-ray tube with a W target (Magpro TUB00155, Moxtek; maximum working voltage 70 kV, maximum power 12 W) is used as the X-ray source. The dose rate of this X-ray tube is calibrated by a Radcal Accu-Gold X-ray dosimeter with a 10×6-180 model ion chamber. The I-V traces and I-t curves under X-ray irradiation were also recorded using the 6517B high-precision electrometer (Keithley, USA).

Calculations of Sensitivity(S)[2]: S is defined as the collected charge per unit area under X-ray irradiation and can be determined by

$$S = \frac{I_{X-ray} - I_{dark}}{D \times A} \tag{3}$$

where $I_{X\text{-}ray}$ and I_{dark} are the currents recorded under X-ray irradiation and in the dark, respectively, D is the irradiation dose rate, and A is the effective area of the detector.

Calculations of Signal-to-Noise Ratio (SNR)[2]: SNR can be calculated by

$$SNR = \frac{I_{signal}}{I_{noise}} \tag{4}$$

where I_{signal} is the signal current and is derived by subtracting the average $I_{X-ray}(\overline{I_{X-ray}})$ by the average dark current $(\overline{I_{dark}})$ and I_{noise} is the noise current and is obtained by calculating the standard deviation of the I_{X-ray}.

$$I_{signal} = \overline{I_{X-ray}} - \overline{I_d} \tag{5}$$

$$I_{noise} = \sqrt{\sum_{1}^{n} \left(I_i - \overline{I_{X-ray}}\right)^2} \tag{6}$$

Dark current drift[3] (I_{drift}) is calculated by

$$I_{drift} = \frac{I_t - I_0}{(E \times t \times A)} \tag{7}$$

where the I_t and I_0 are, respectively, dark current at time t and 0, E represents the electric field, and A is the device area.

Density functional theory (DFT) simulations: all DFT simulations were performed through the VASP software within the plane augmented wave (PAW) framework.[4-6] The reciprocal space was sampled using a uniform, Γ-centered grid with a spacing of 0.30/2π $Å^{-1}$ between k points. The simulated systems were the (*R*-MBA)$_2$PtI$_6$ enantiomer (the S was not simulated, as it is expected to have the same electronic structure as its mirror image) and the racemic (MBA)$_2$PtI$_6$. The crystal structure of the latter has molecules disordered over two positions, one with a greater occupation (0.68) than the other (0.32). For the simulations, we select the molecular geometry with the greatest occupation. Two types of simulations were performed. To gain a qualitative understanding of the electronic structure (DOS, band structure, and charge density distribution) and its relationship with the crystal structure, simulations were performed at the experimental geometry, adopting the PBE functional[7] and neglecting spin-orbit coupling (SOC). In this case, the plane-wave energy cutoff was set to 500 eV. These are referred to as "experimental geometry PBE simulations" in this document. Another set of simulations was performed to quantitatively analyze the energy levels within and across the two crystal structures. For these, the crystal geometry was relaxed to the nearest energy minimum by adopting the above-mentioned settings and augmenting them with Grimme's D2 dispersion correction[8] to account for weak intermolecular forces that play an important role in the cohesion in this crystal. HSE06[9] simulations, including SOC with a plane-wave energy cutoff of 400 eV, were then performed at the PBE-D2 optimized geometry. These simulations are referred to in this document as "relaxed geometry HSE simulations". During structural relaxation, the self-consistent field (SCF) and geometry optimization procedures were stopped when the energy difference between two successive steps was lower than 10^{-4} eV. For electronic structure evaluation, the SCF threshold was lowered to 10^{-5} eV.

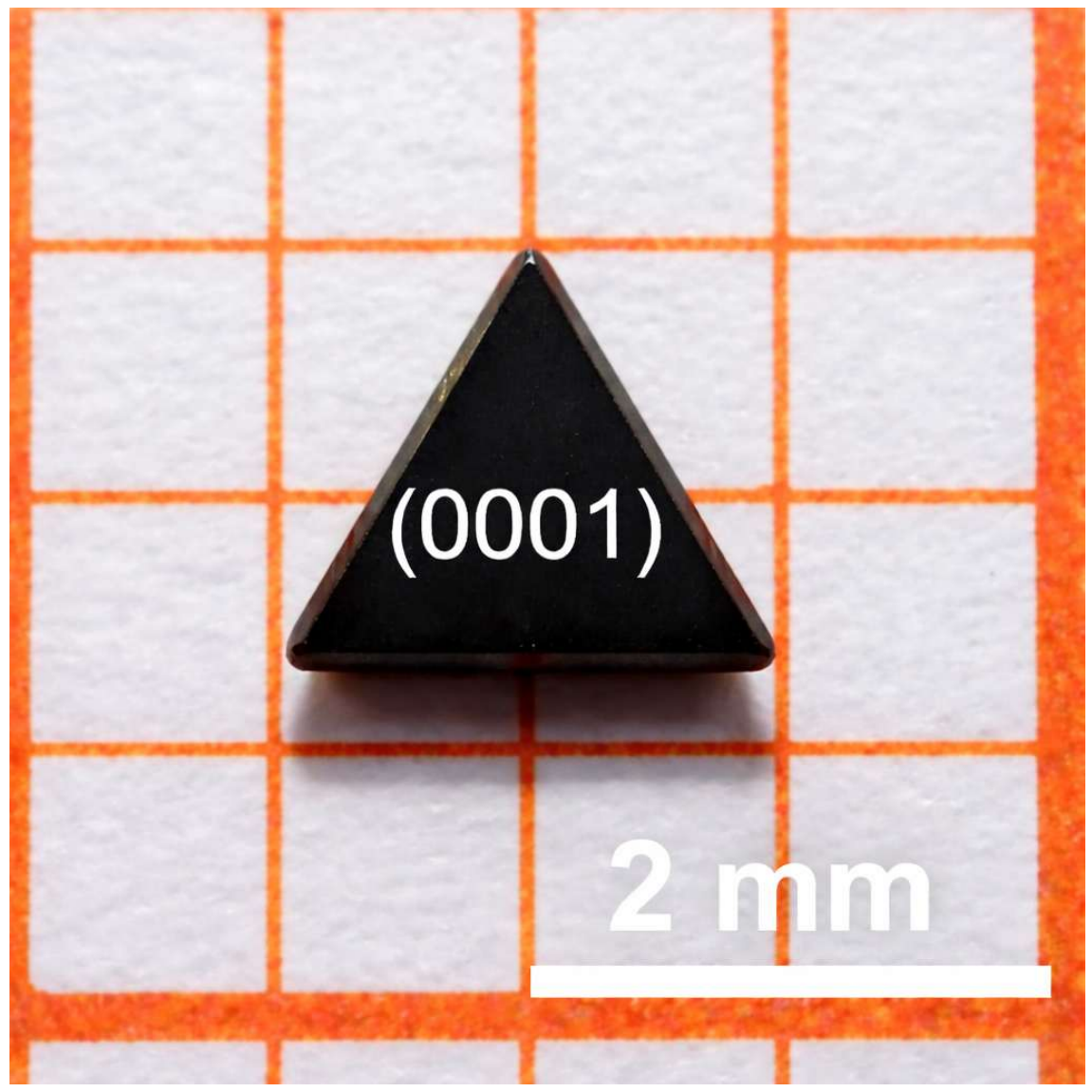

Figure S1. Photograph of the SC sample of (R/S-MBA)$_2$PtI$_6$.

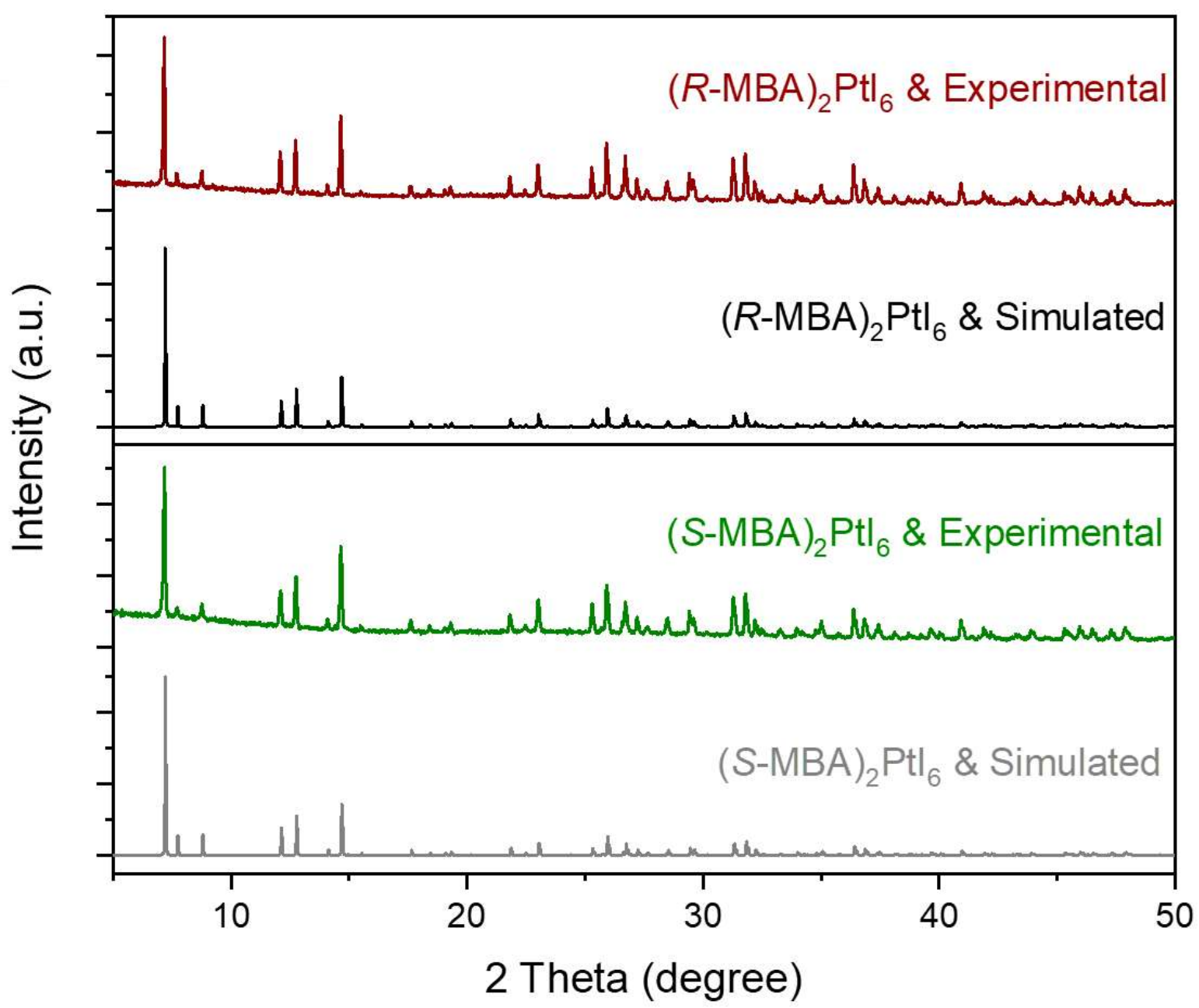

Figure S2. Simulated and experimental PXRD patterns of $(R/S\text{-MBA})_2PtI_6$.

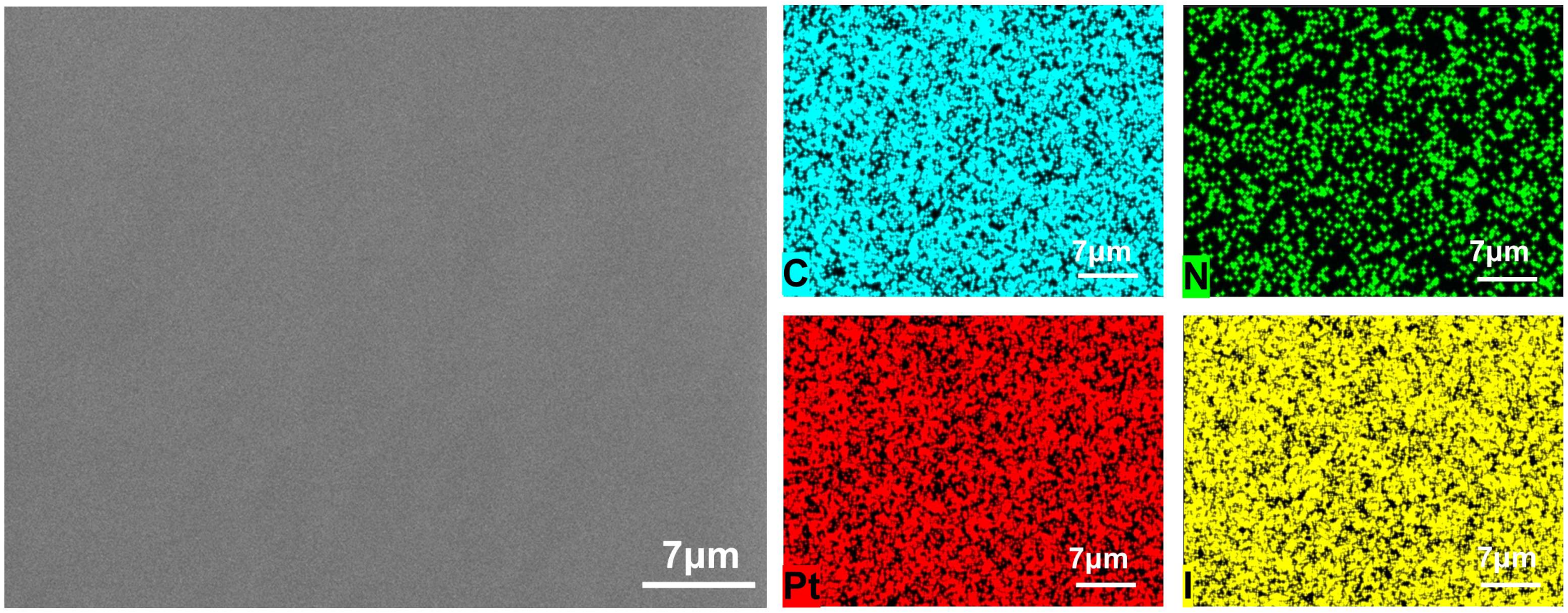

Figure S3. SEM images of the SC surface and EDS element mappings for C, N, Pt, and I elements.

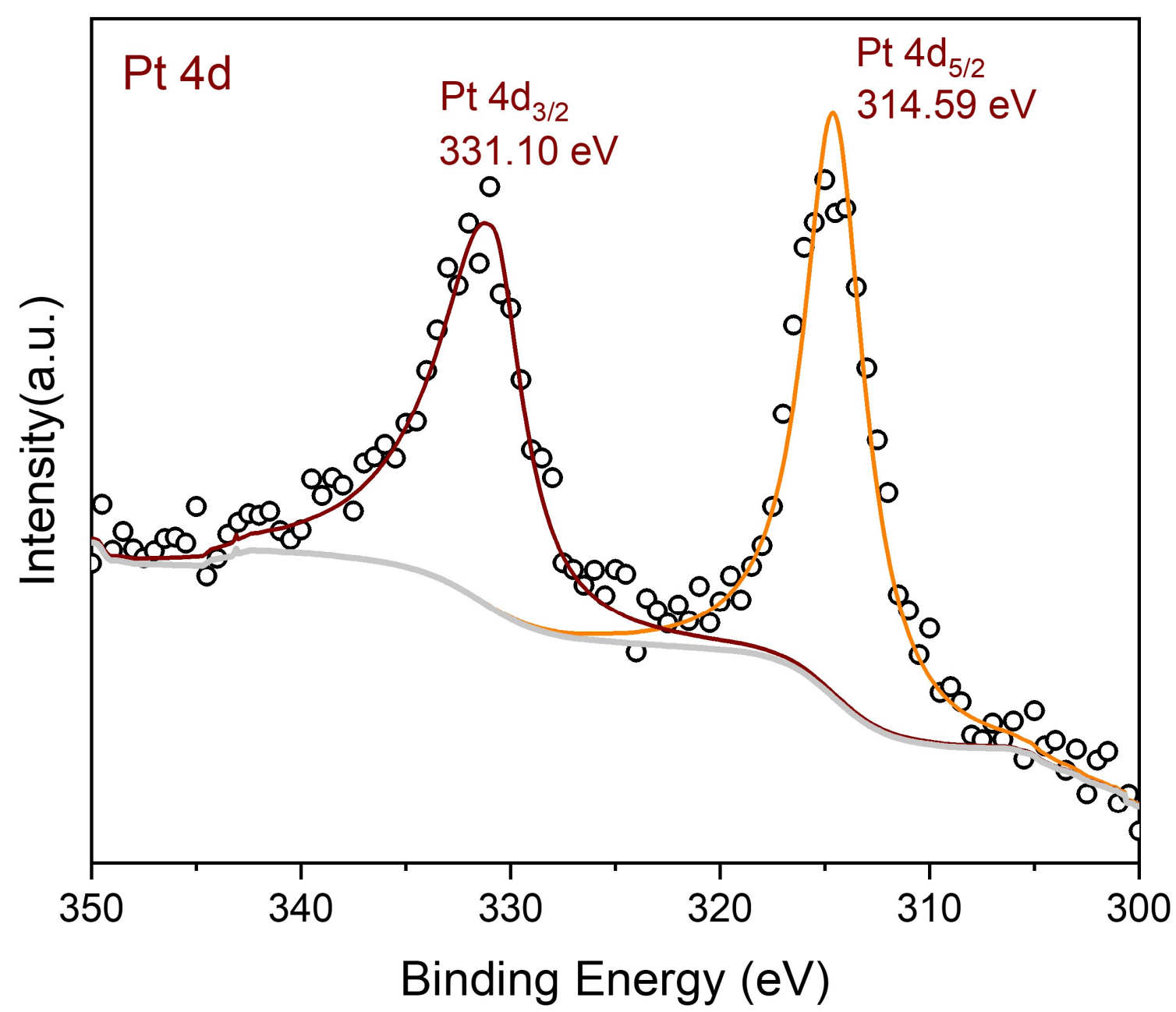

Figure S4. XPS spectra of the Pt 4d spectra of $(R/S\text{-MBA})_2PtI_6$.

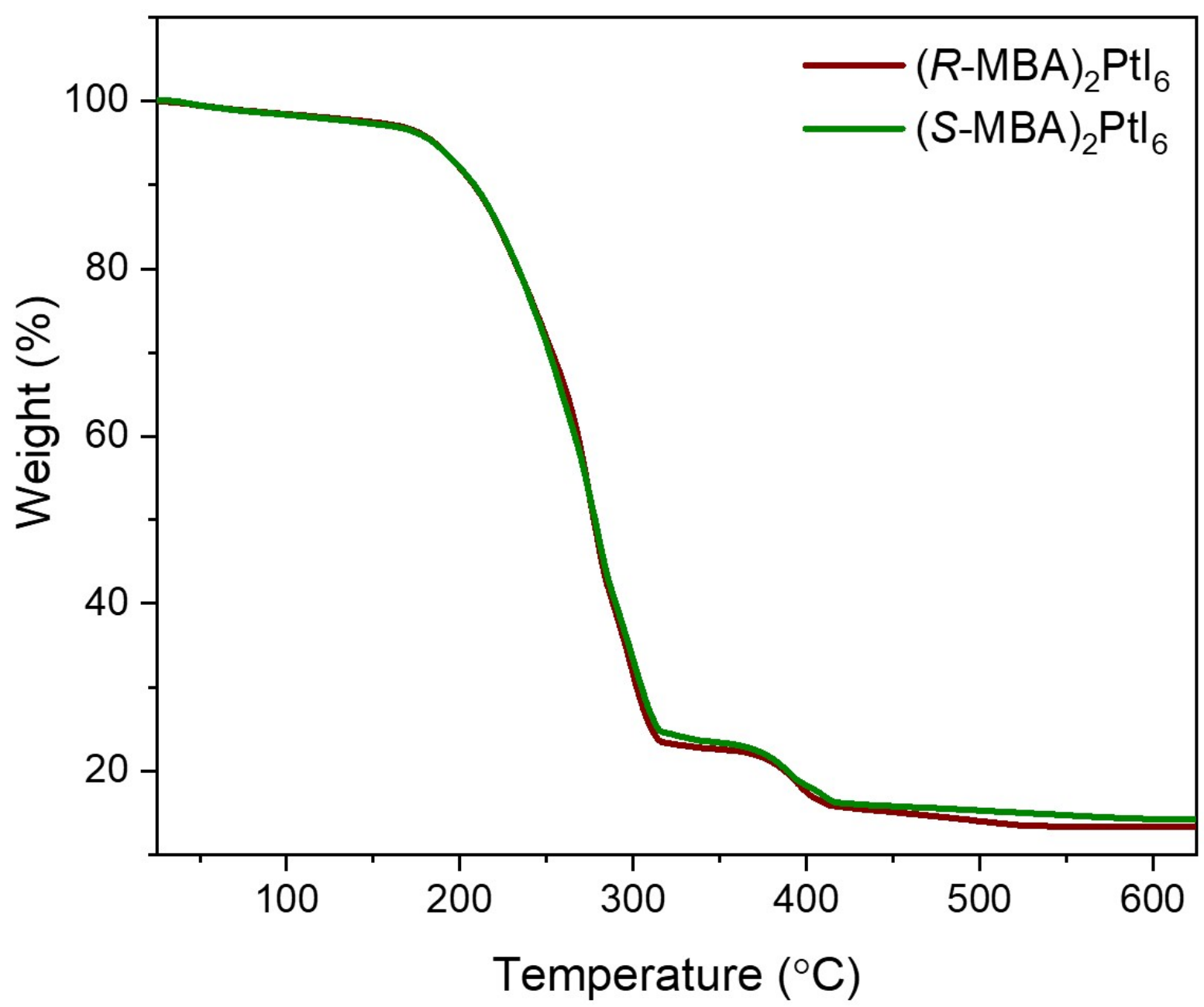

Figure S5. TG curves of $(R/S\text{-MBA})_2PtI_6$ SCs.

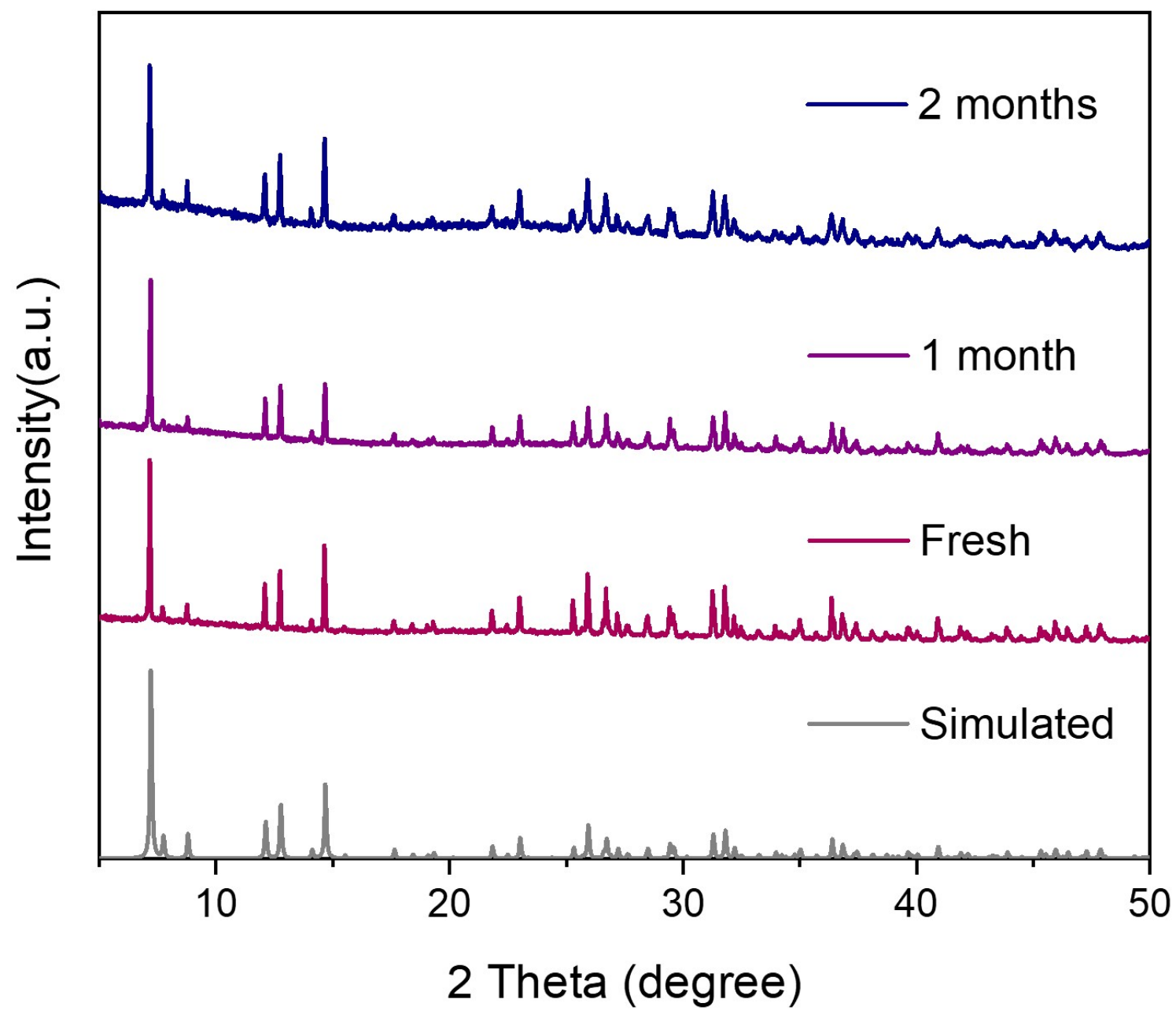

Figure S6. XRD patterns of $(R/S\text{-MBA})_2PtI_6$ samples before and after their exposure to ambient air for two months.

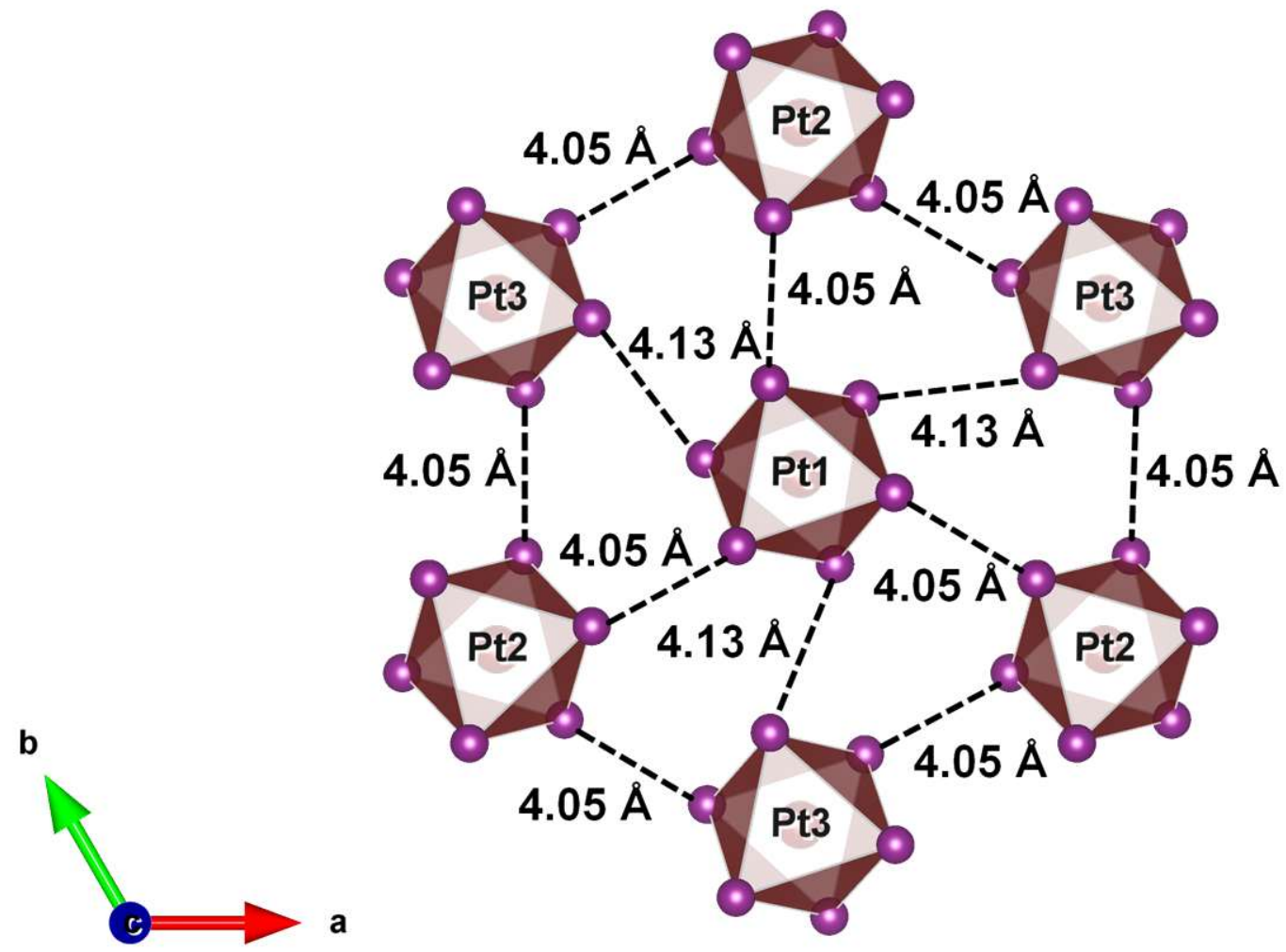

Figure S7. Packing of the inorganic sublayer viewed along the c axis, showing the distance between adjacent octahedra.

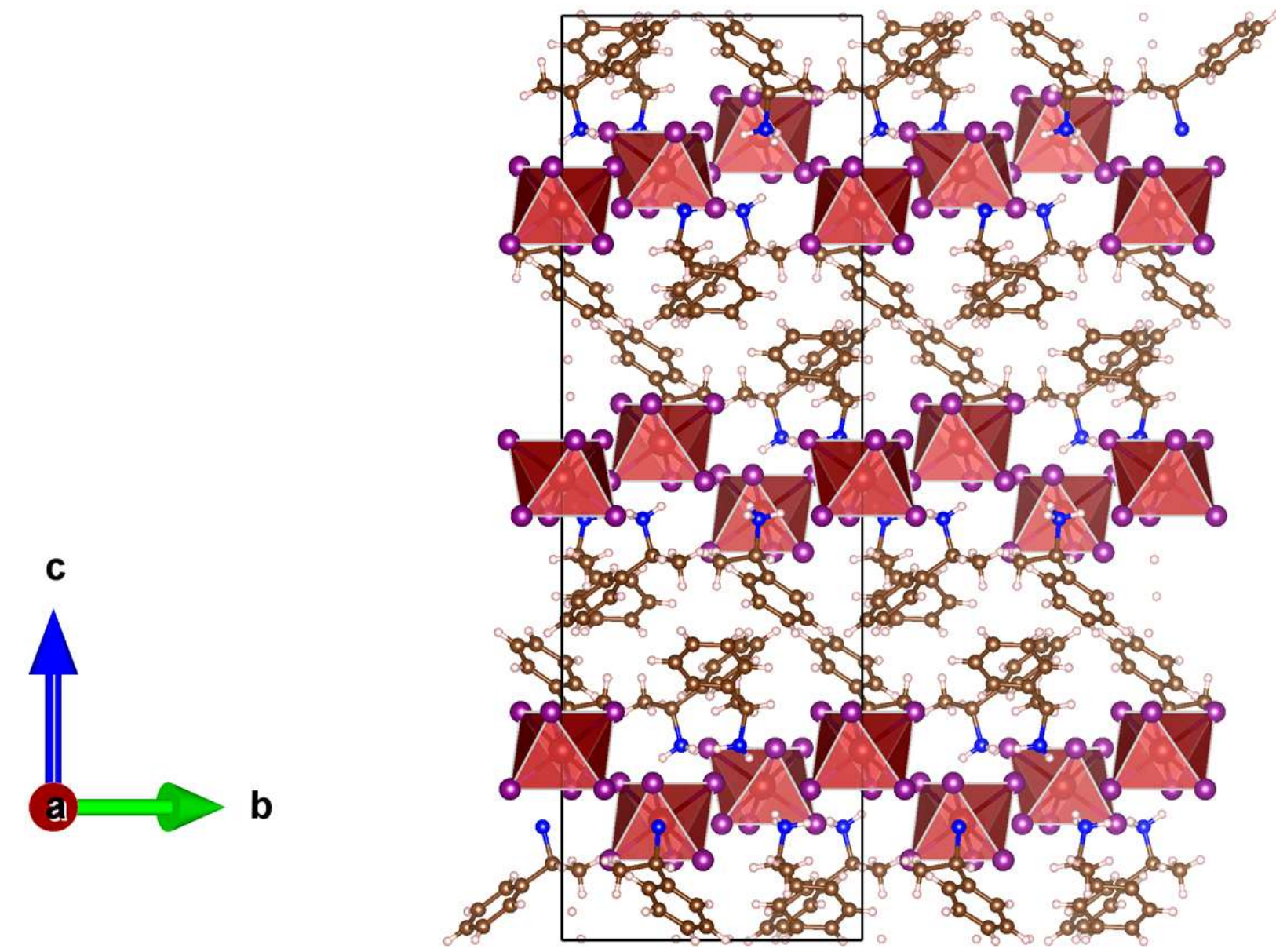

Figure S8. Single crystal structure of $(rac\text{-MBA})_2PtI_6$.

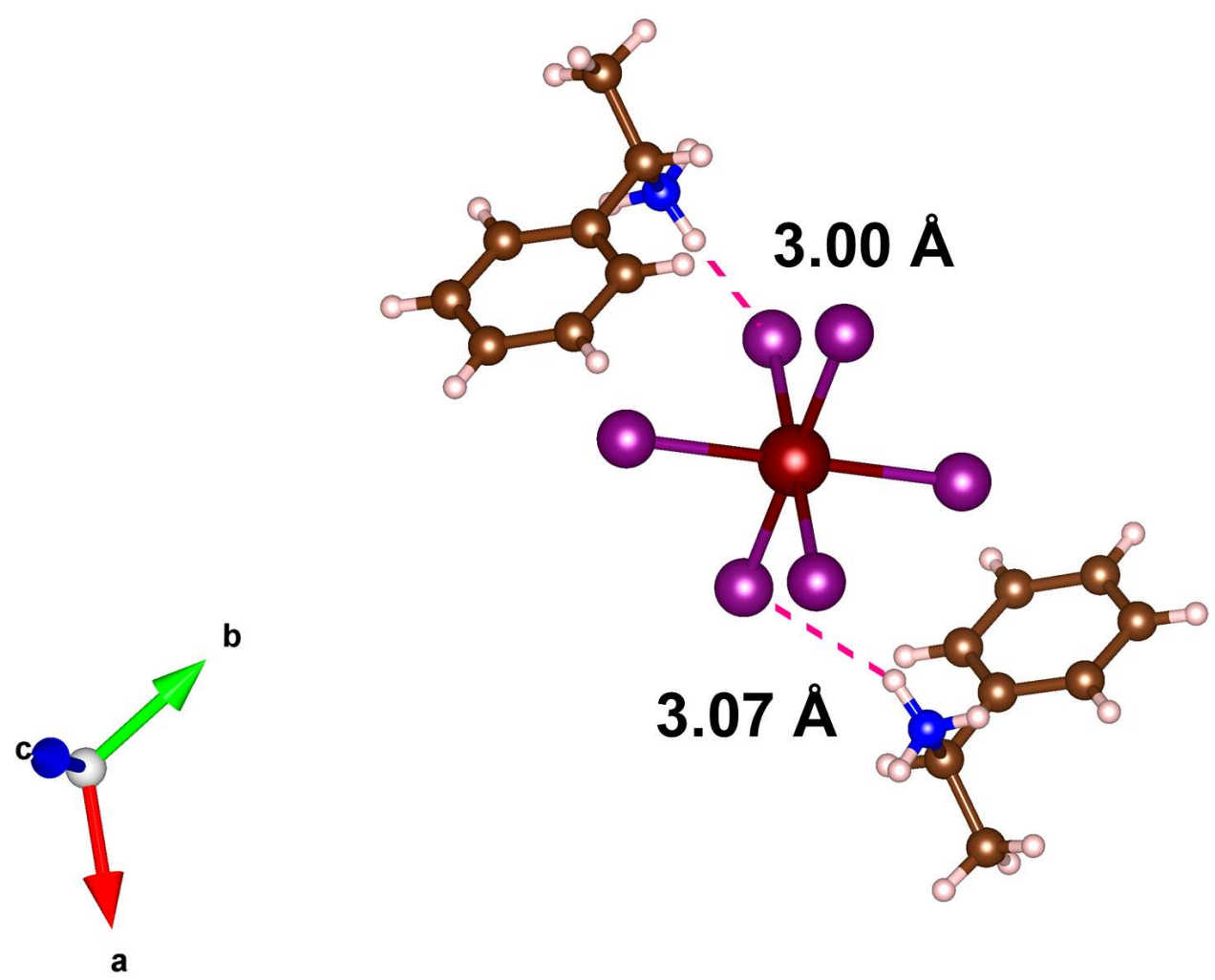

Figure S9. Hydrogen bond distributions around $[Pt(1)I_6]^{2-}$ octahedra in (*rac*-MBA)$_2$PtI$_6$. The other equivalent hydrogen bonds are omitted.

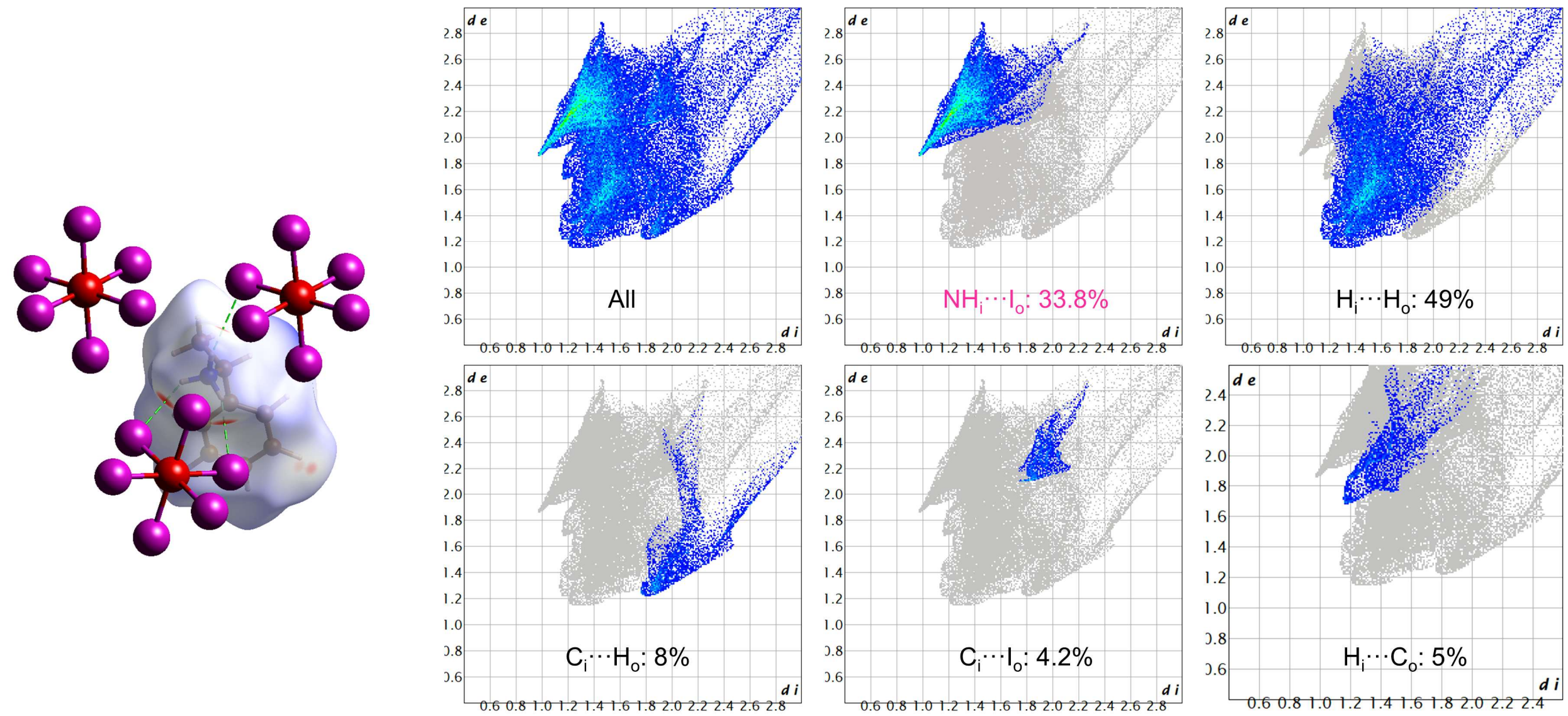

Figure S10. Hirshfeld surface of the *R*-MBA$^+$ cation and corresponding 2D fingerprint maps in $(R\text{-MBA})_2PtI_6$.

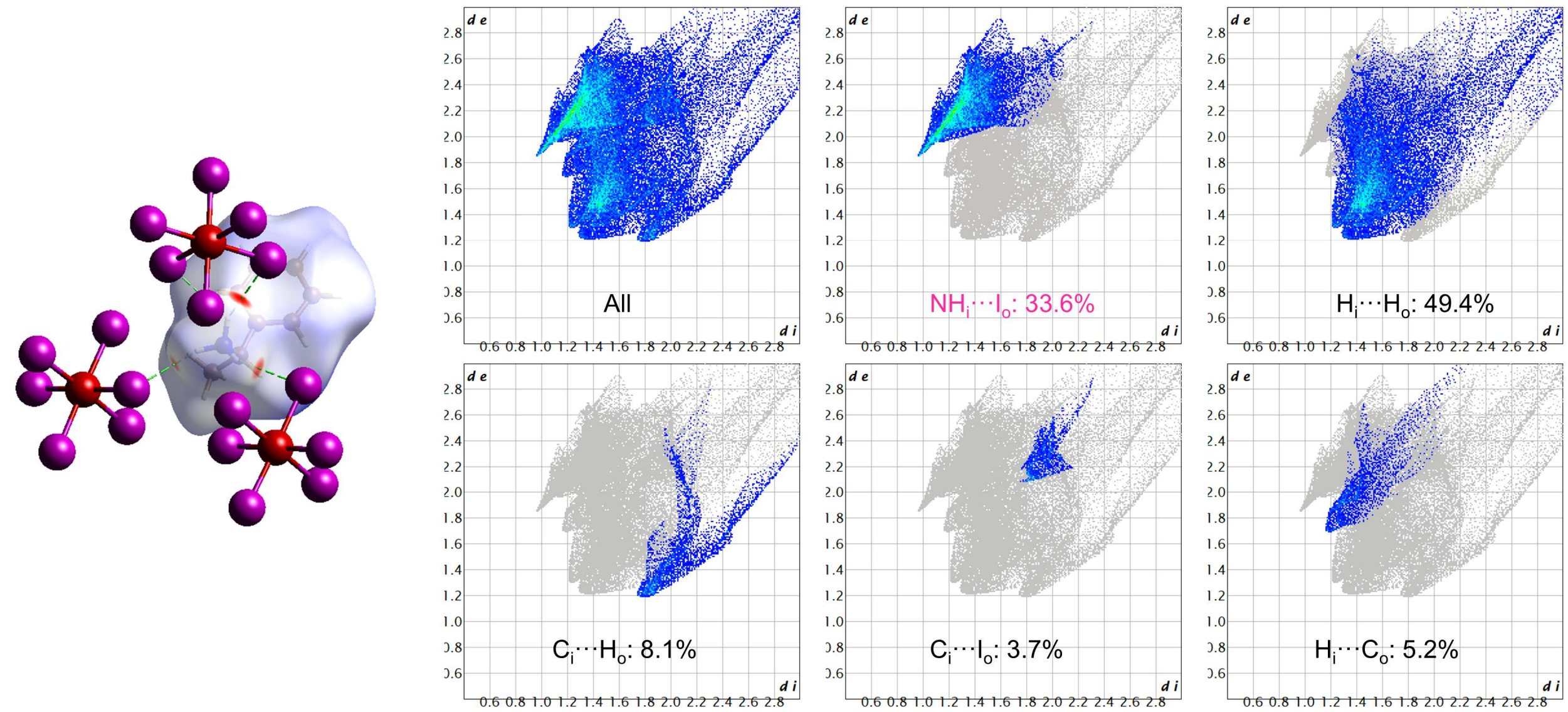

Figure S11. Hirshfeld surface of the *S*-MBA$^+$ cation and corresponding 2D fingerprint maps in $(S\text{-MBA})_2PtI_6$.

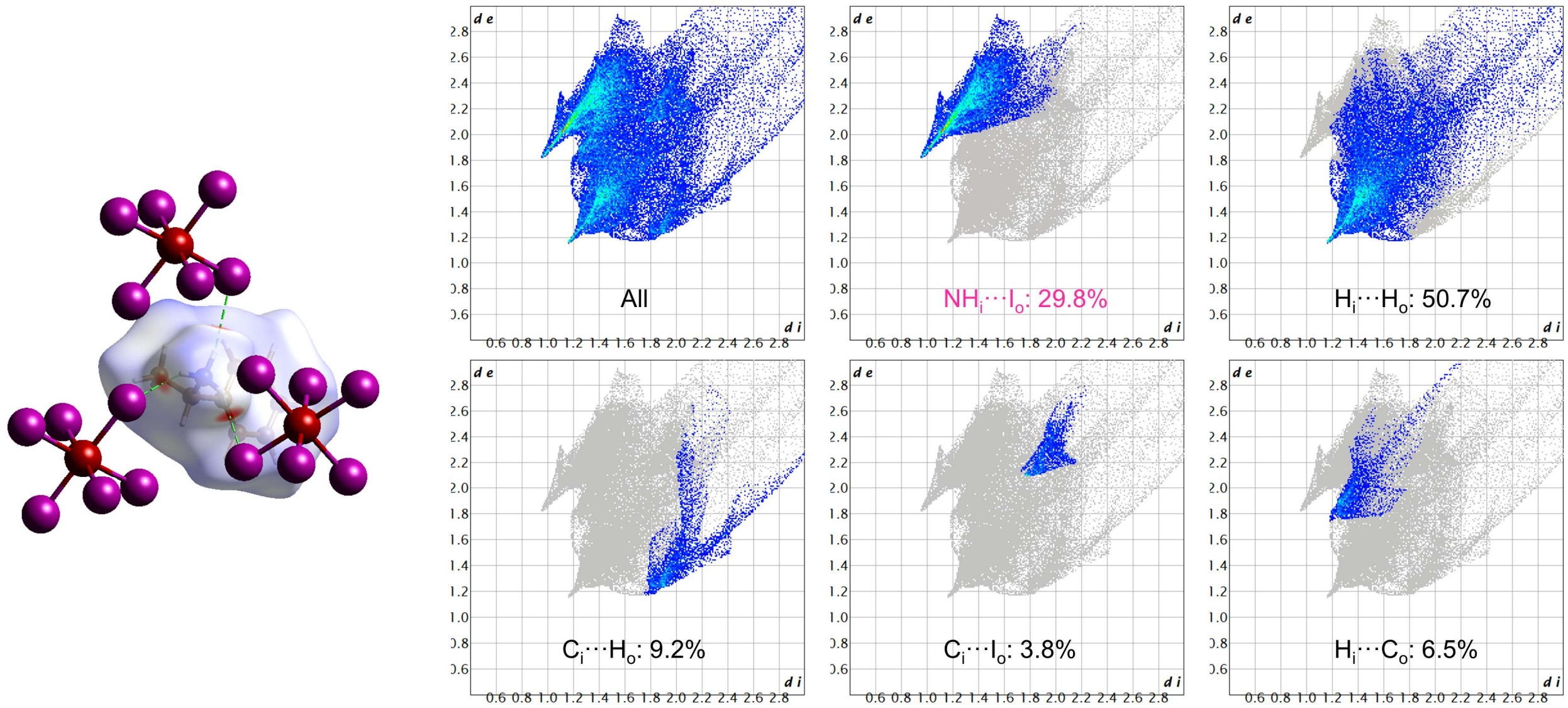

Figure S12. Hirshfeld surface of the *rac*-MBA$^+$ cation and corresponding 2D fingerprint maps in (*rac*-MBA)$_2$PtI$_6$.

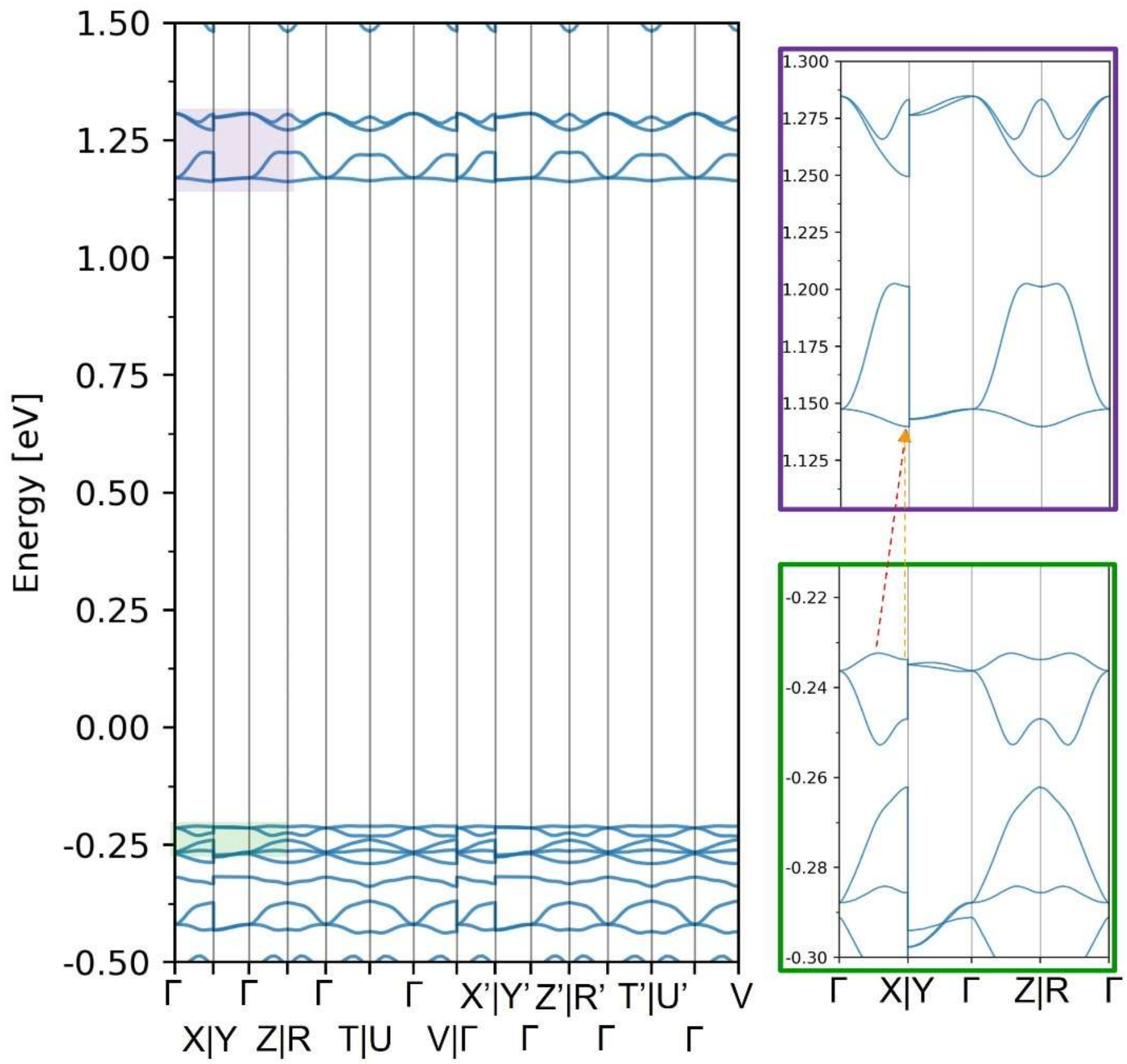

Figure S13. Band structure of the (R-MBA)$_2$PtI$_6$ enantiomer at experimental geometry (experimental geometry PBE simulations). The enlargements of valence band maximum (green) and conduction band minimum (violet) are shown on the right, with red and orange arrows indicating the indirect and direct band gaps, respectively.

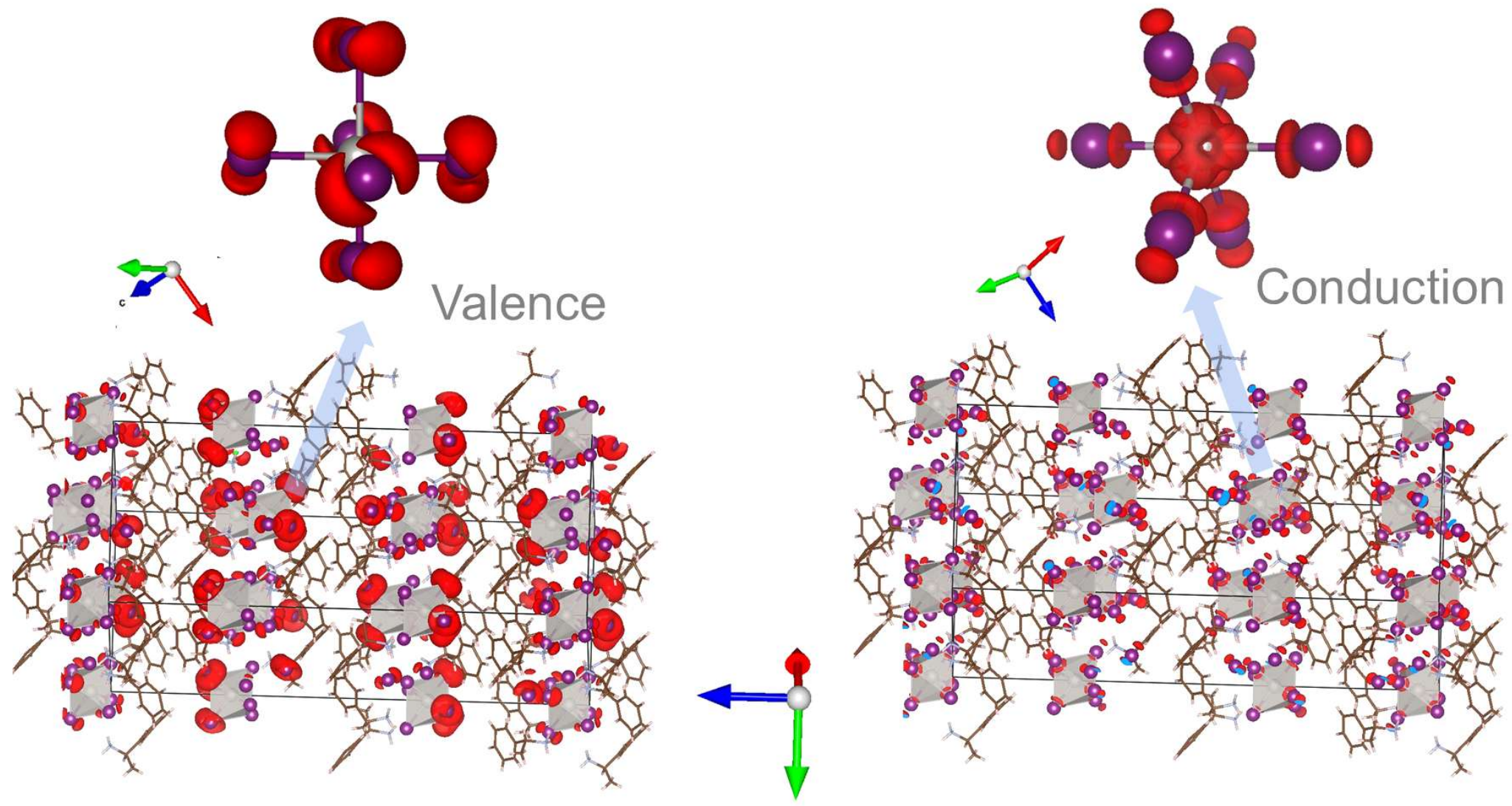

Figure S14. Partial charge density of (R-MBA)$_2$PtI$_6$. Charge density distributions of the valence (-0.4 eV to the Fermi level) and conduction (+1.0 to +2.0 eV) bands are shown as red isosurfaces.

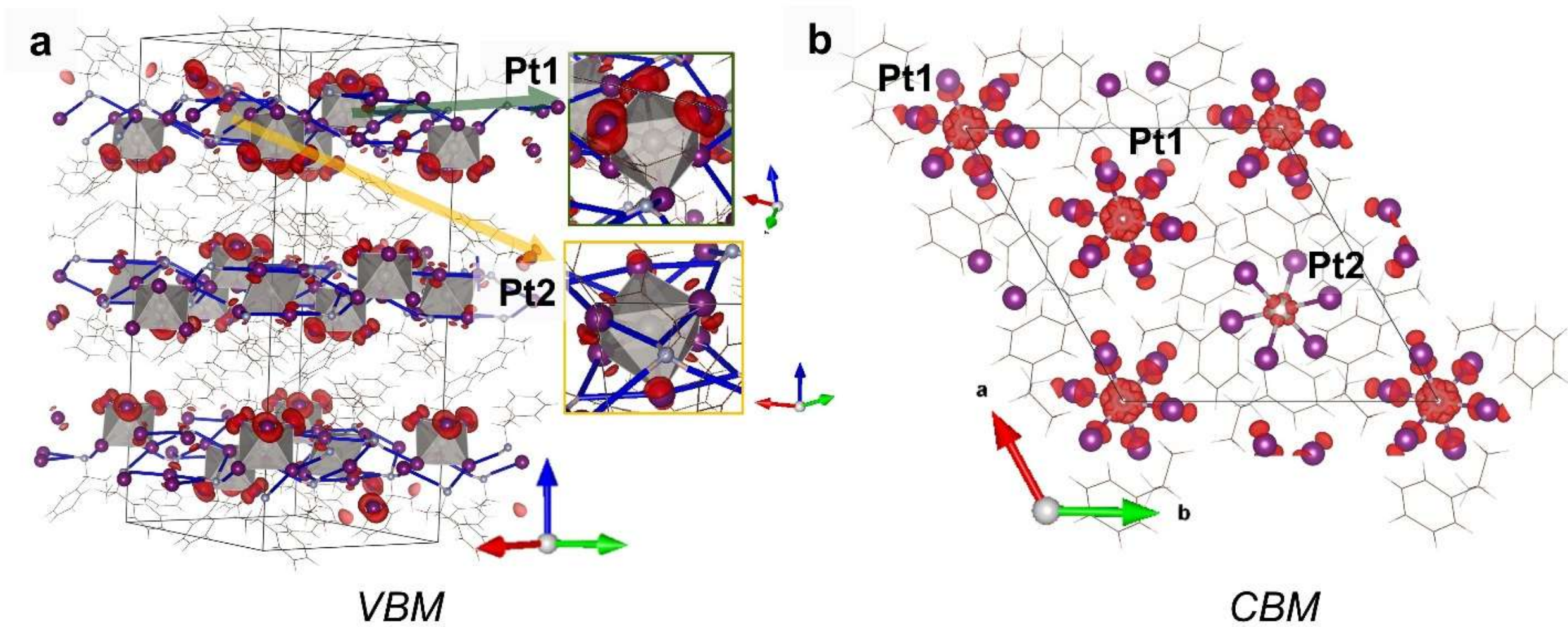

Figure S15. Electron density distribution, shown as red isosurfaces, of the valence band maximum (a) and conduction band minimum (b) for (*rac*-MBA)$_2$PtI$_6$. Energy ranges with respect to the Fermi level: VMB=-0.4/0.0 eV, CBM=0.5/1.3 eV. The labels for symmetry-independent atoms are the same as in **Tables S2-S8**. In MBA molecules, C-C and C-H bonds are shown as thin lines for clarity. In panel a, N atoms are explicitly shown, and N-H···I hydrogen bonds (HBs) are shown as blue thick lines (all H···I contacts within 3.2 Å are considered as HBs). In panel b, only part of the unit cell is shown for clarity, which, however, contains all symmetry-independent PtI$_6$ octahedra. Note how the valence band is localized on those I atoms that do not accept HBs, and how the dominant contribution of the conduction band is found on PtI$_6$ octahedra with the lowest distortion index σ^2 (*cfr.* **Table S8**).

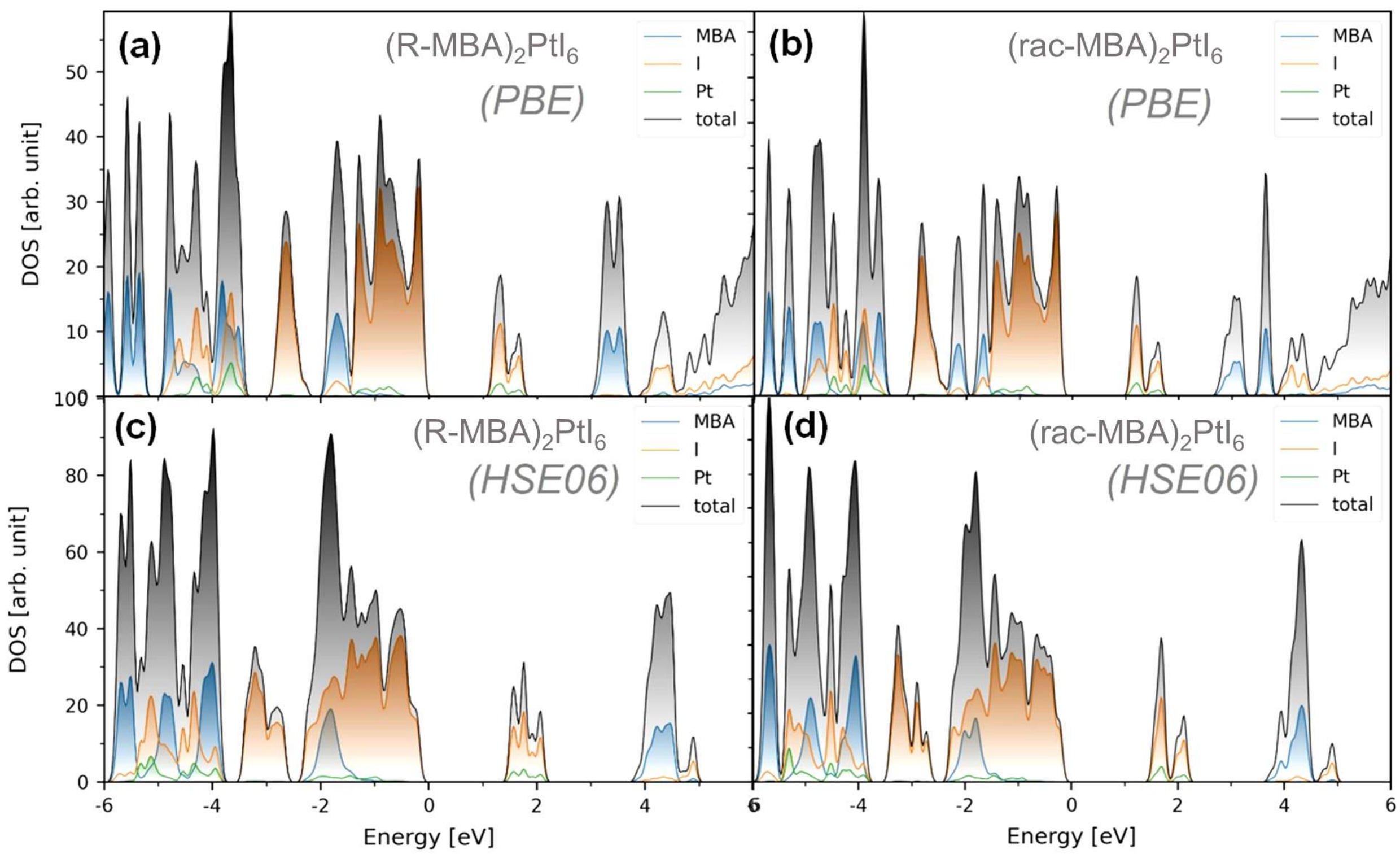

Figure S16. Density of states (DOS) of (*R*-MBA)$_2$PtI$_6$ (a,c) and (*rac*-MBA)$_2$PtI$_6$ (b,d) at the experimental geometry (PBE functional, no SOC; a,b) and optimized geometry (HSE06 functional+SOC; b,d).

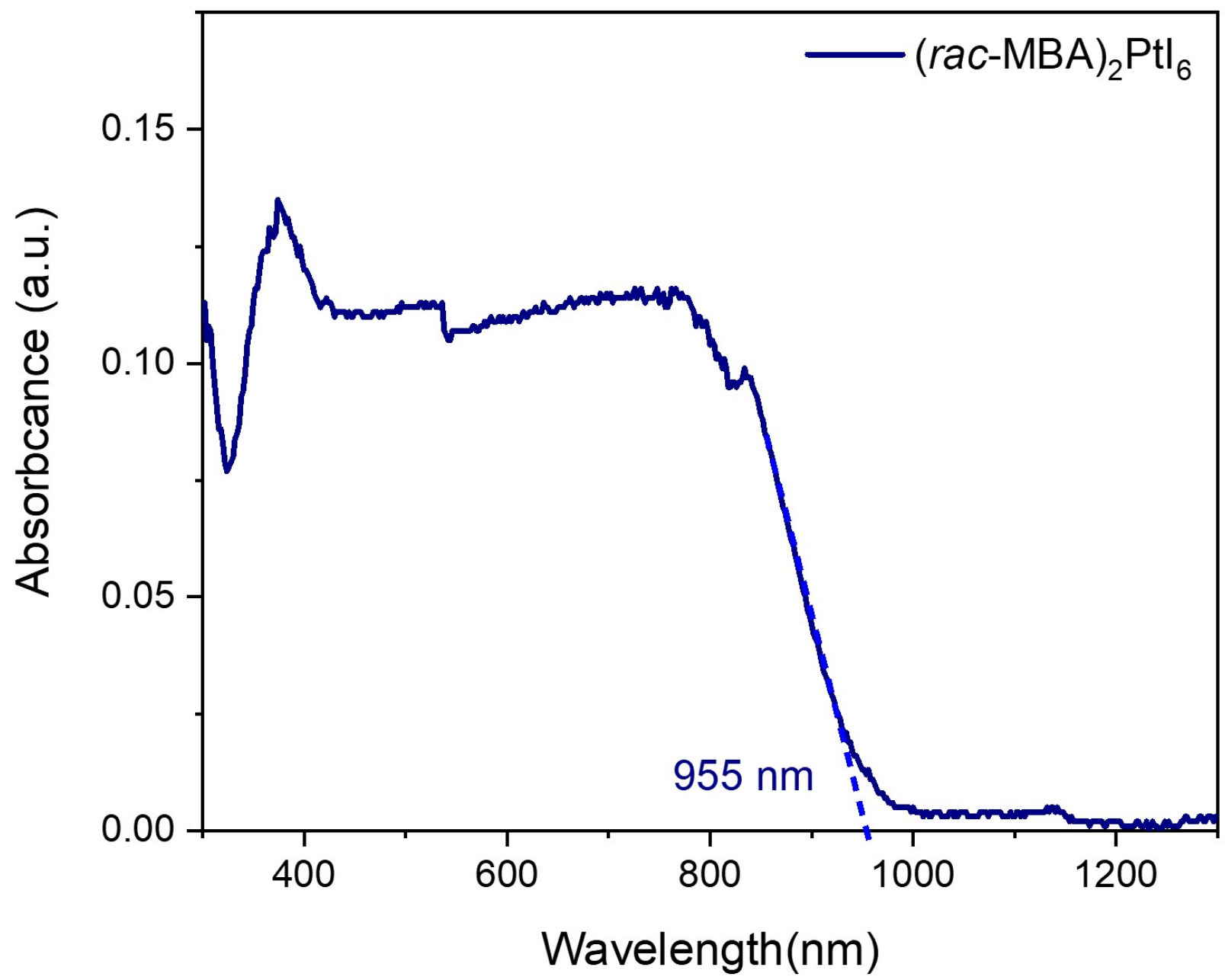

Figure S17. UV-Vis-NIR absorption spectra of (rac-MBA)$_2$PtI$_6$ single crystals.

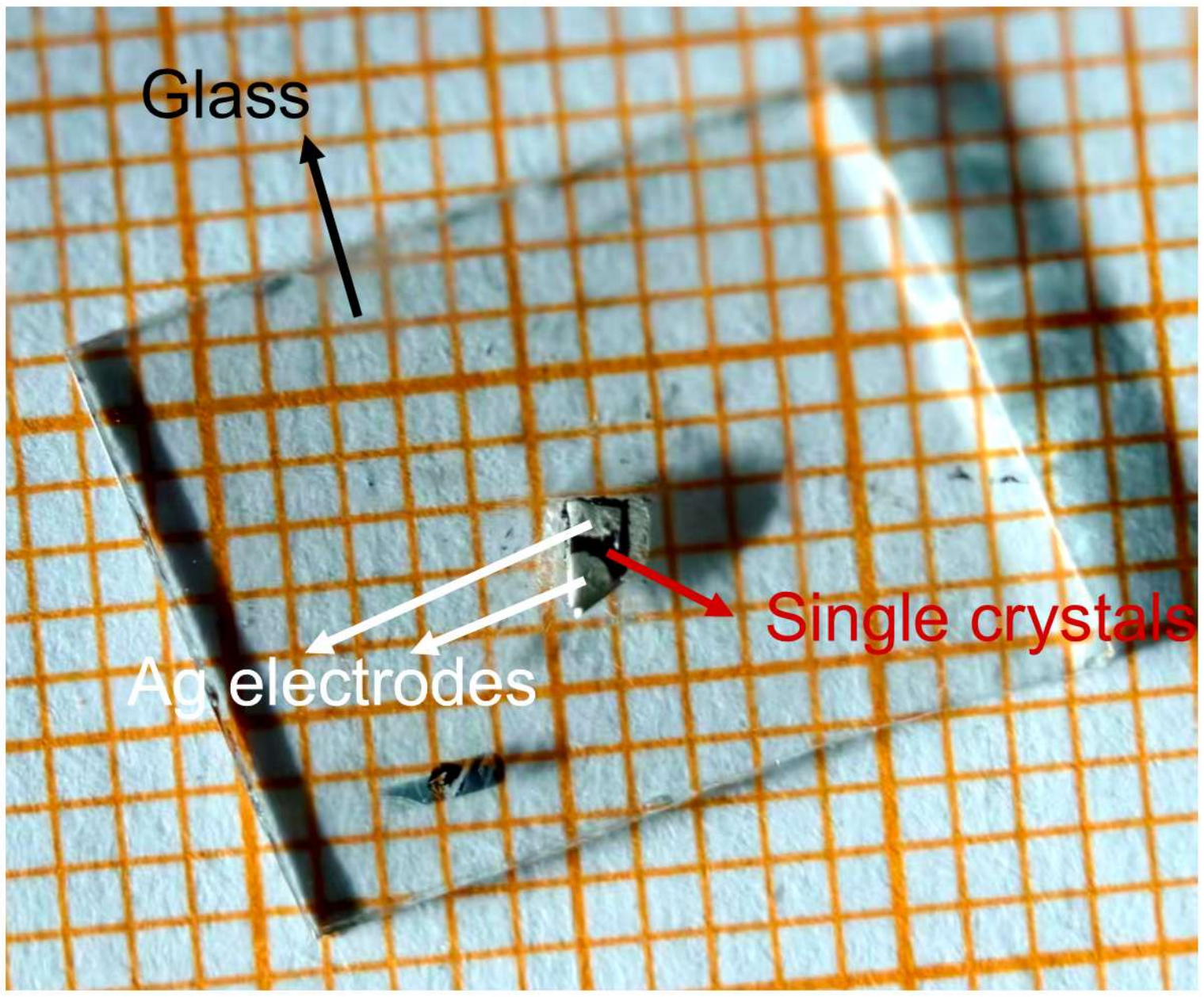

Figure S18. Photograph the planar device based on (R-MBA)$_2$PtI$_6$ SCs.

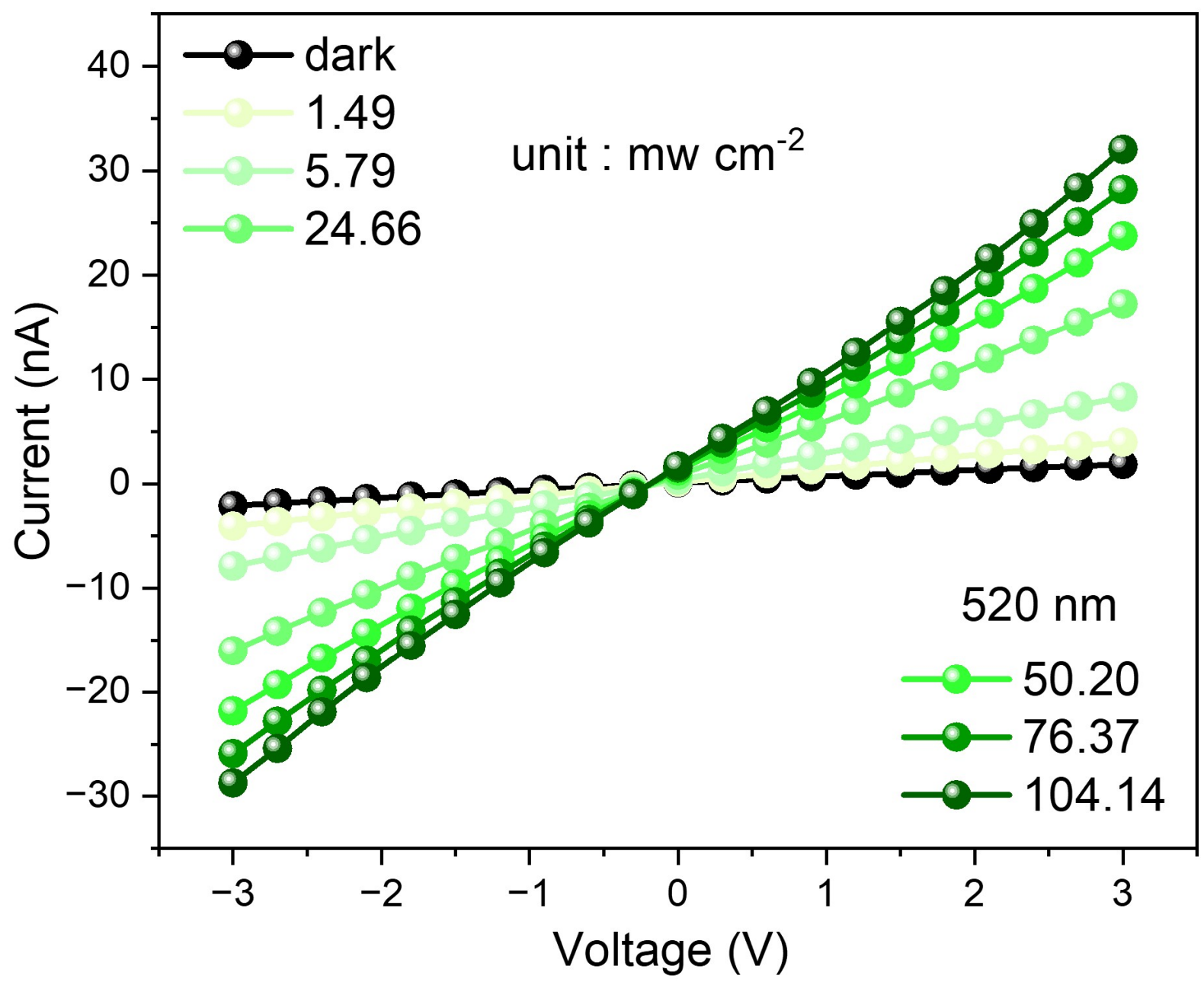

Figure S19. I-V traces of $(R\text{-MBA})_2PtI_6$ PD measured under 520 nm light illumination with different light power densities.

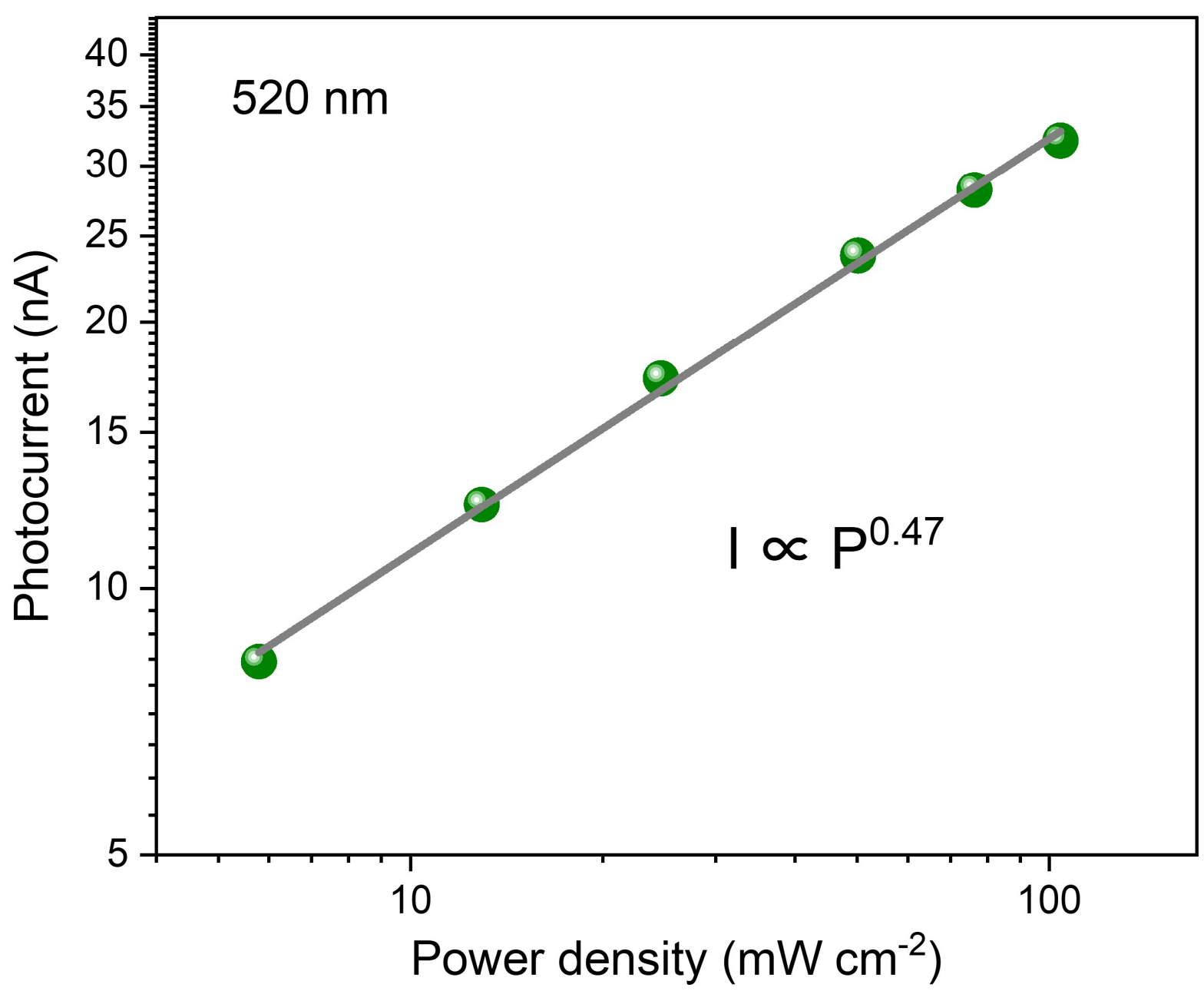

Figure S20. Power density-dependent photocurrent of the (R-MBA)$_2$PtI$_6$ PD at a fixed bias voltage of 3 V under 520 nm illumination.

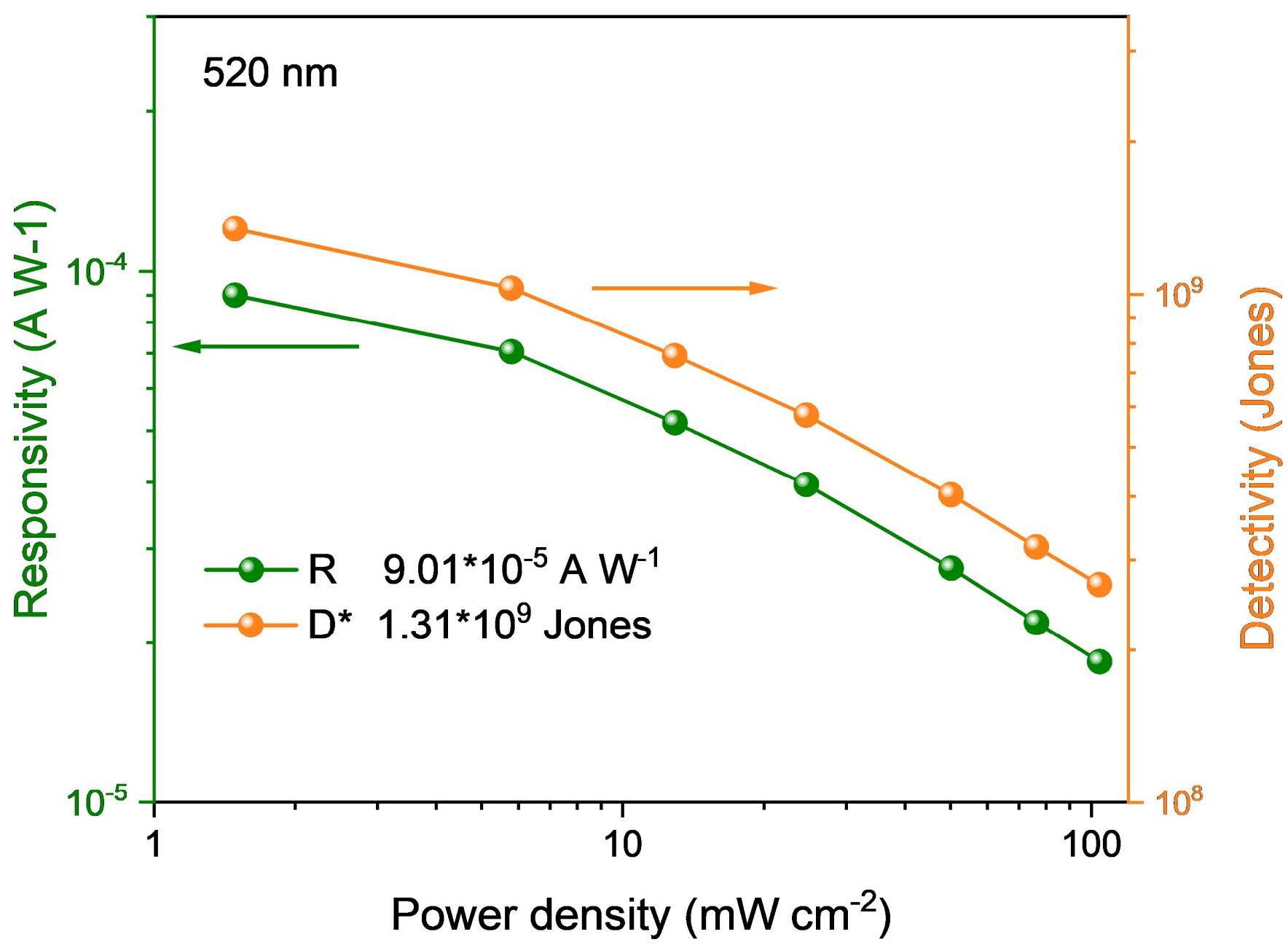

Figure S21. R and D* of $(R\text{-MBA})_2PtI_6$ PD under 520 nm illumination calculated from the photocurrent measured at a 3 V bias.

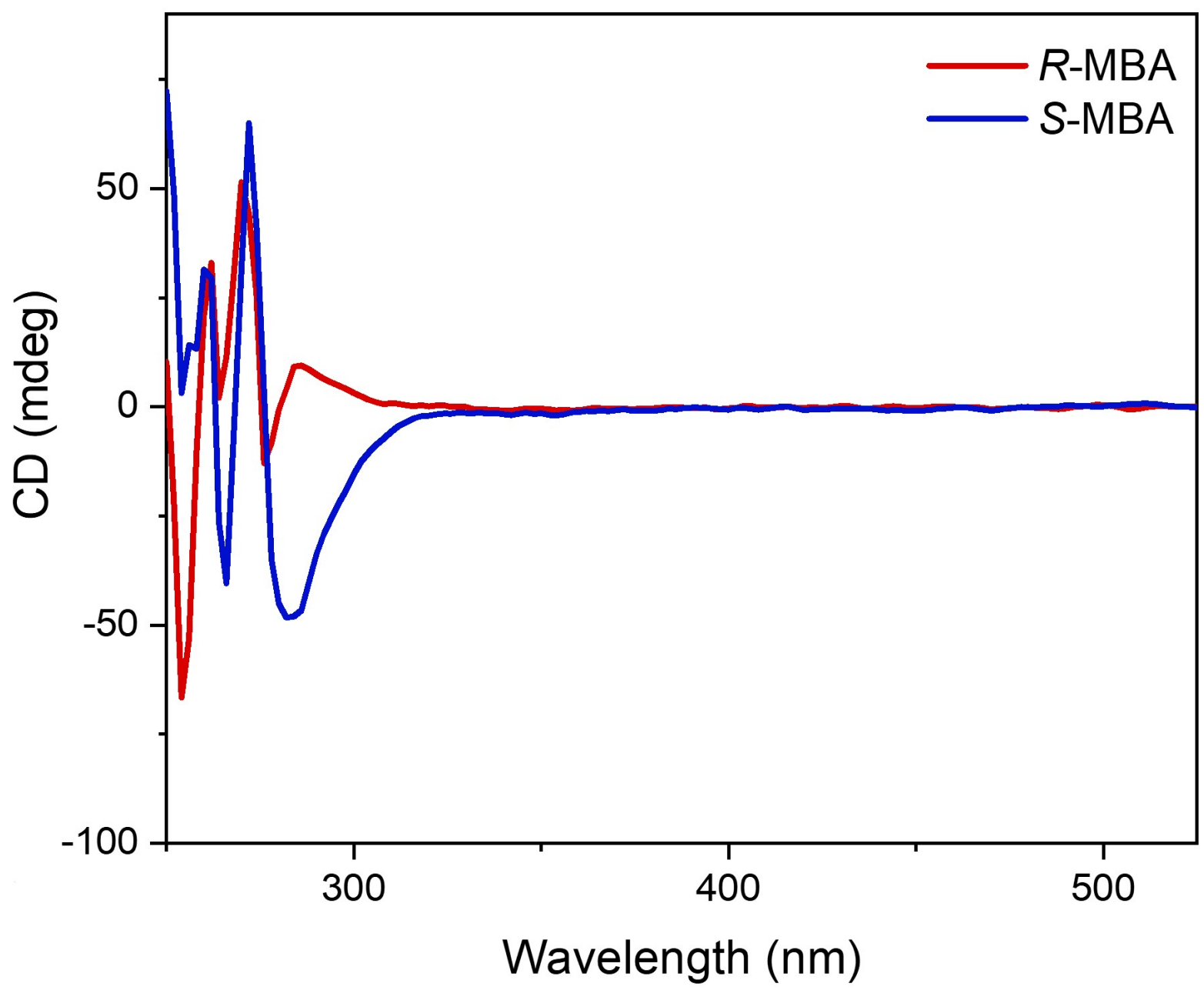

Figure S22. CD spectra of *R*/*S*-MBA diluted in ethanol solution.

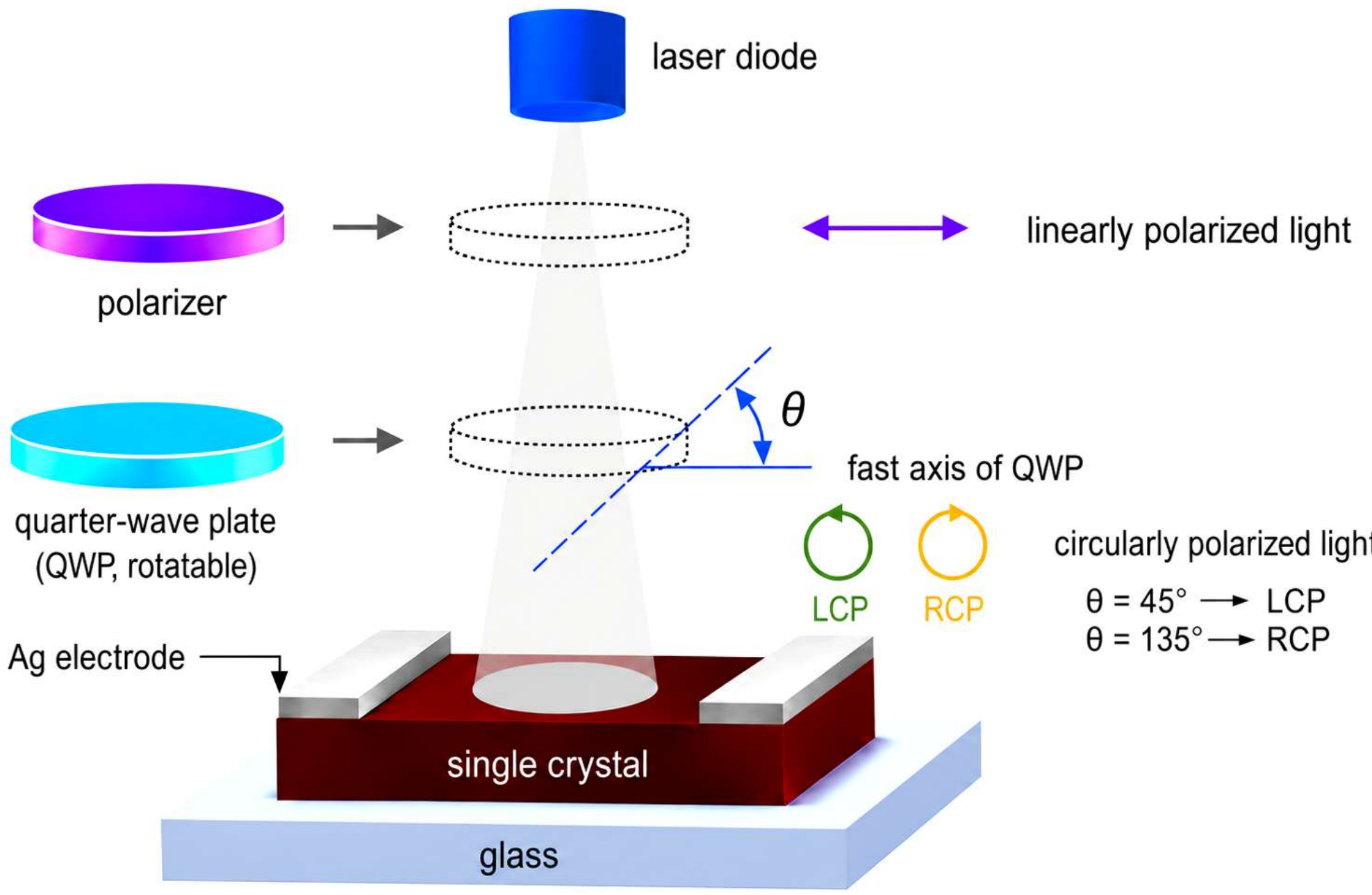

Figure S23. Schematic illustration of the CPL detection based on $(R\text{-MBA})_2PtI_6$ SC.

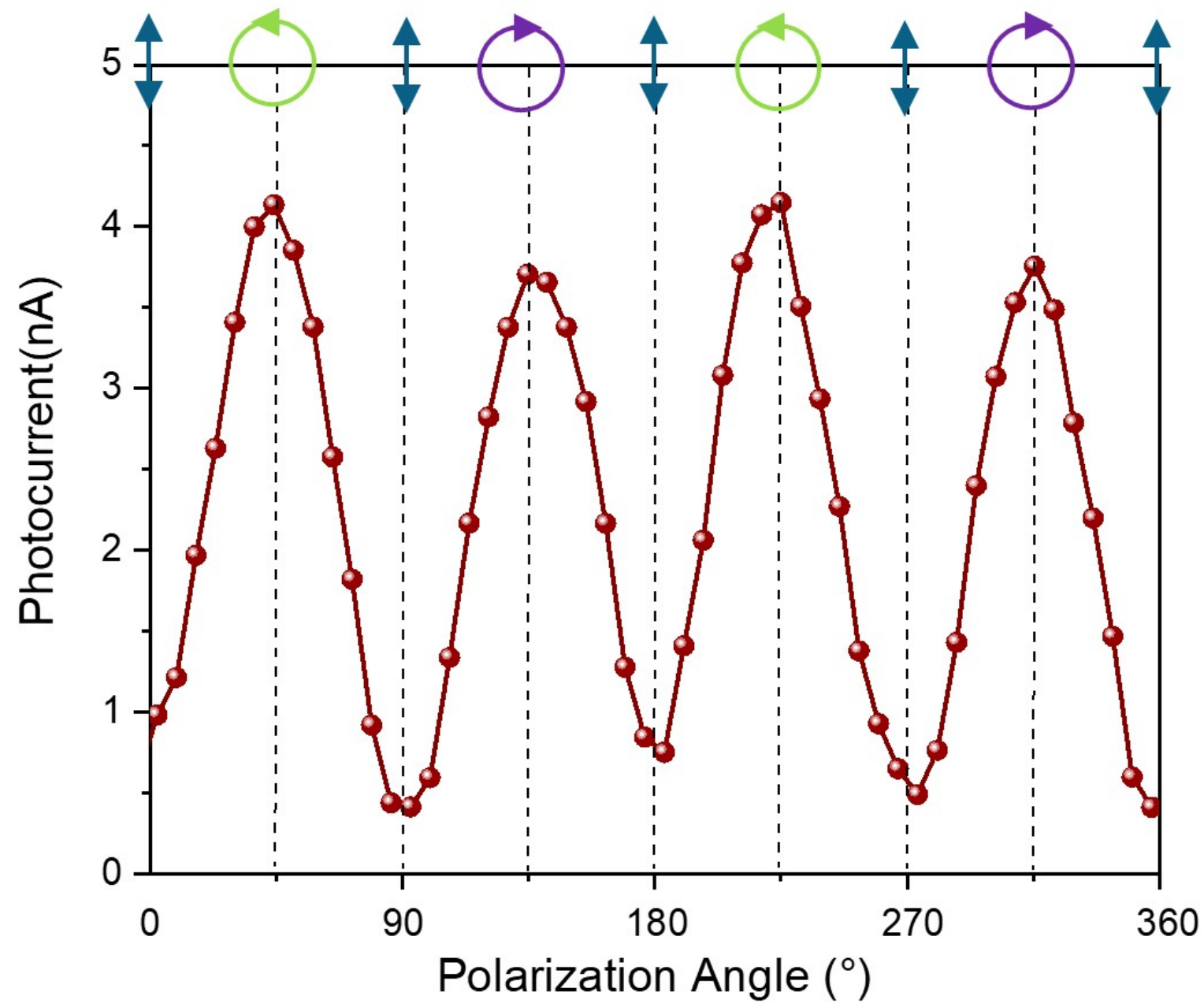

Figure S24. Short-circuit photocurrent of the $(R\text{-}MBA)_2PtI_6$ PD as a function of the rotation angle of the quarter-wave (λ/4) plate at 780 nm.

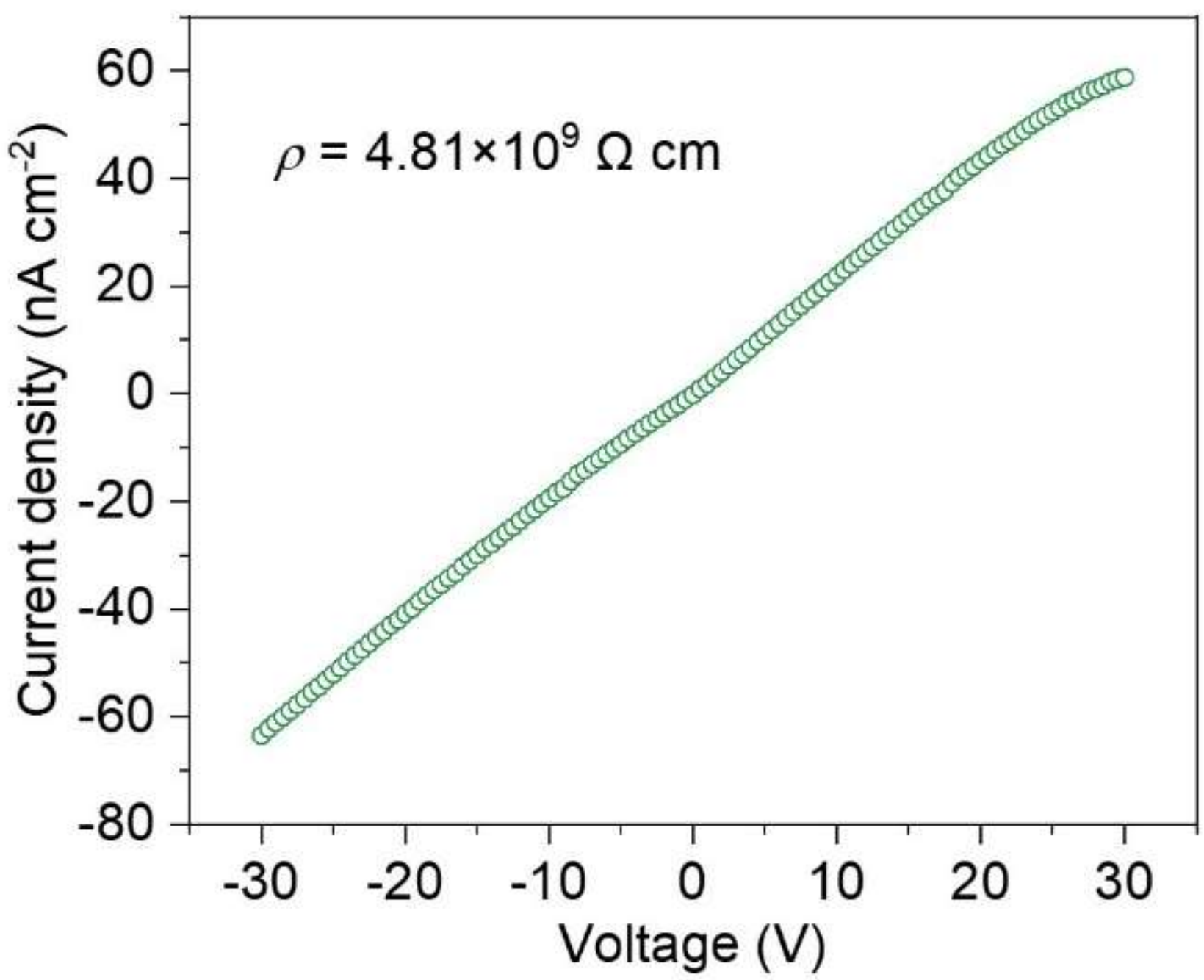

Figure S25. J-V curve of $(R\text{-MBA})_2PtI_6$ SC detector in the dark, yielding a bulk resistivity of 4.81×10^9 Ω cm.

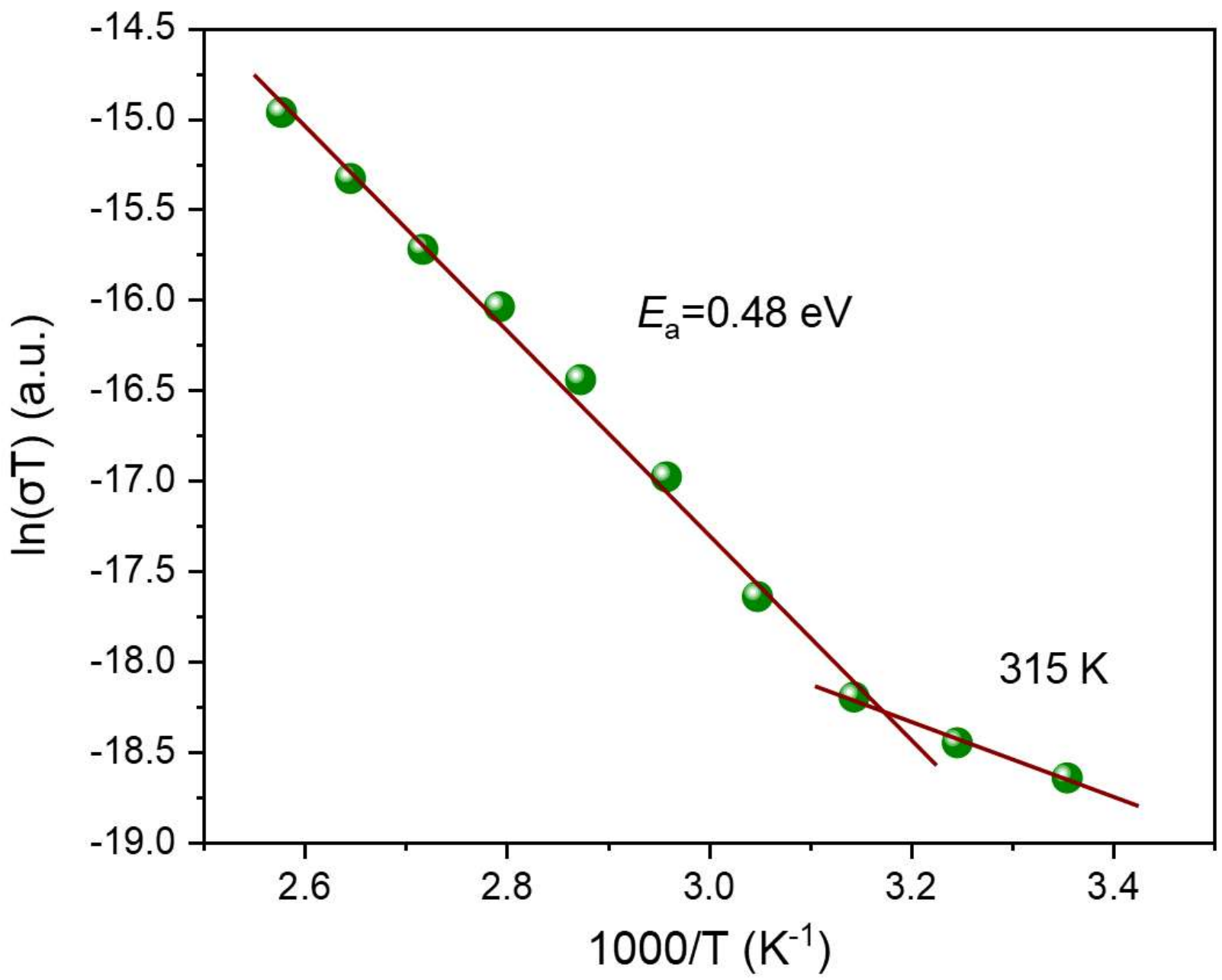

Figure S26. Temperature-dependent conductivity of (R-MBA)$_2$PtI$_6$ SC.

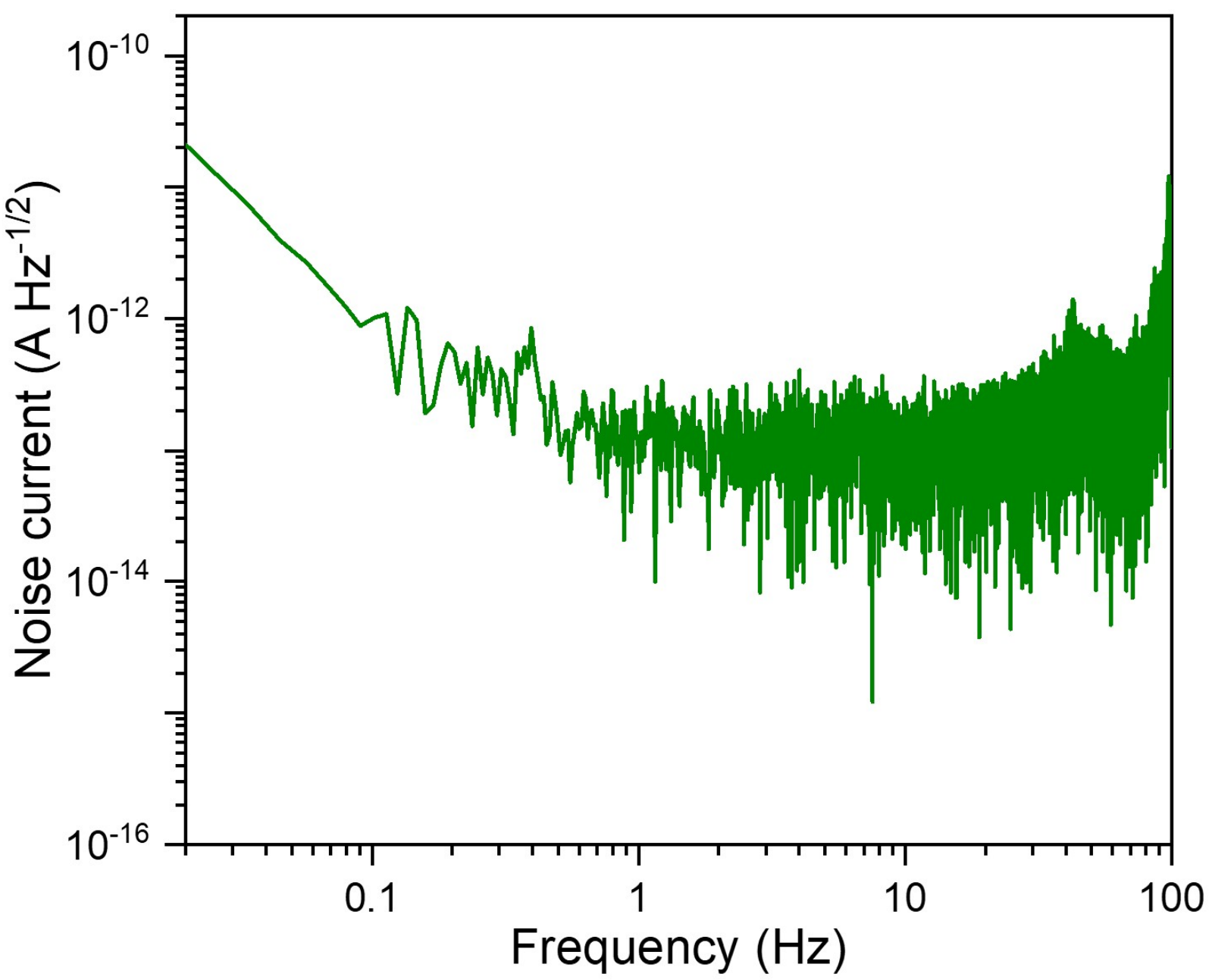

Figure S27. Noise current spectra of (R-MBA)$_2$PtI$_6$ device in the dark.

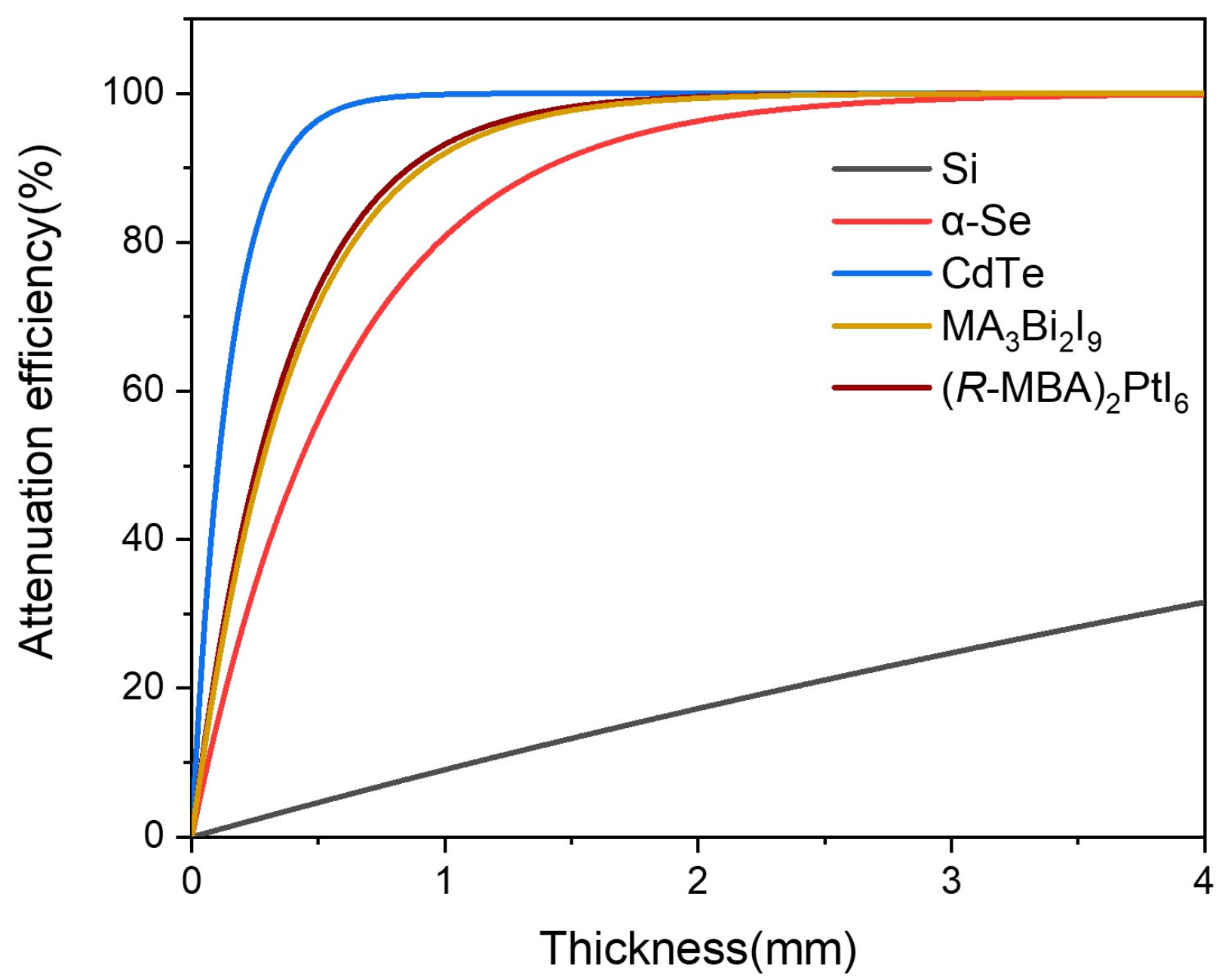

Figure S28. Attenuation efficiency of $(R\text{-MBA})_2PtI_6$ Si, α-Se, CdTe, and $MA_3Bi_2I_9$ for 50-keV X-ray photons versus thickness.

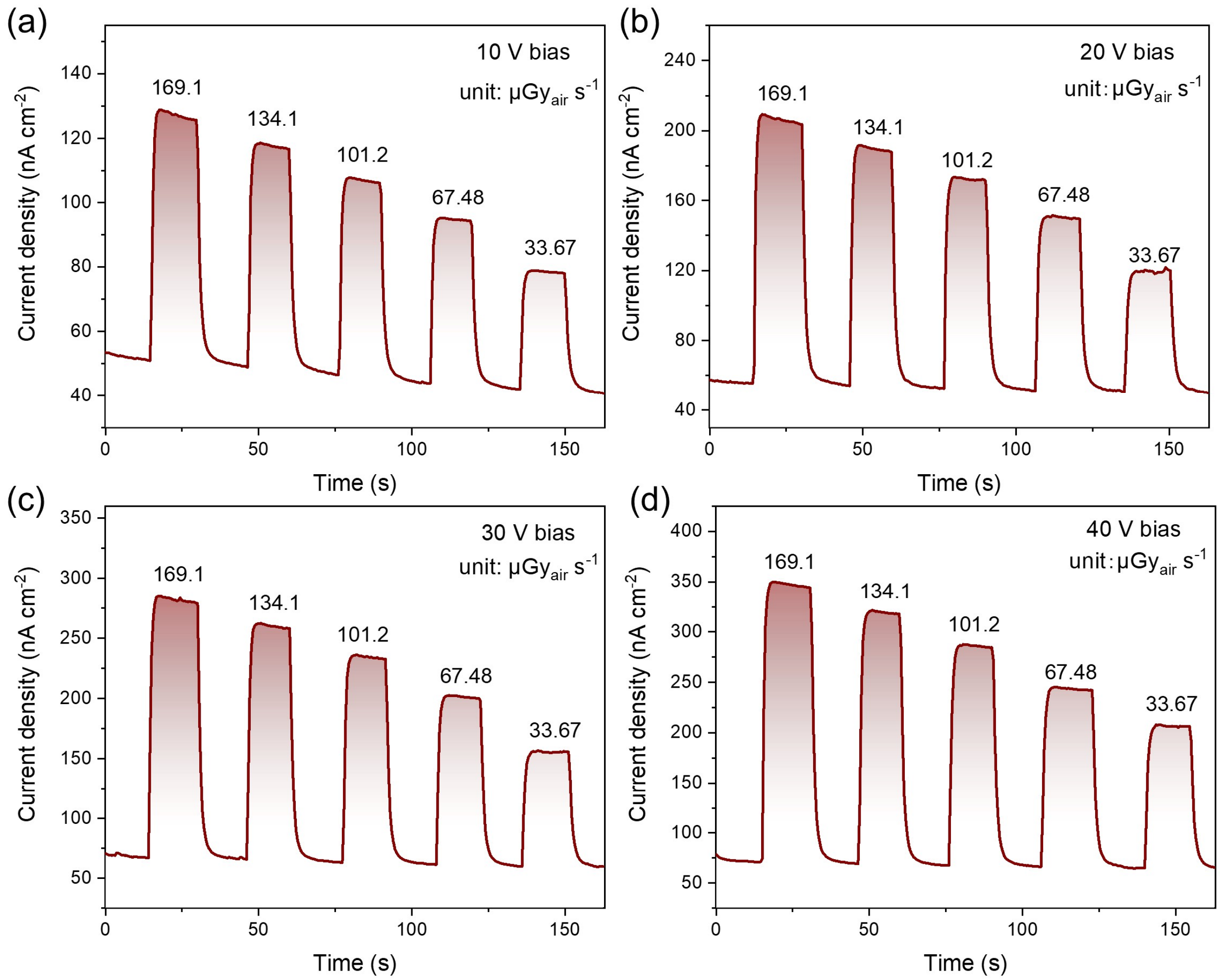

Figure S29. J–T curves of (R-MBA)$_2$PtI$_6$ under X-ray irradiation with different dose rates at a fixed bias voltage (10, 20, 30, and 40 V).

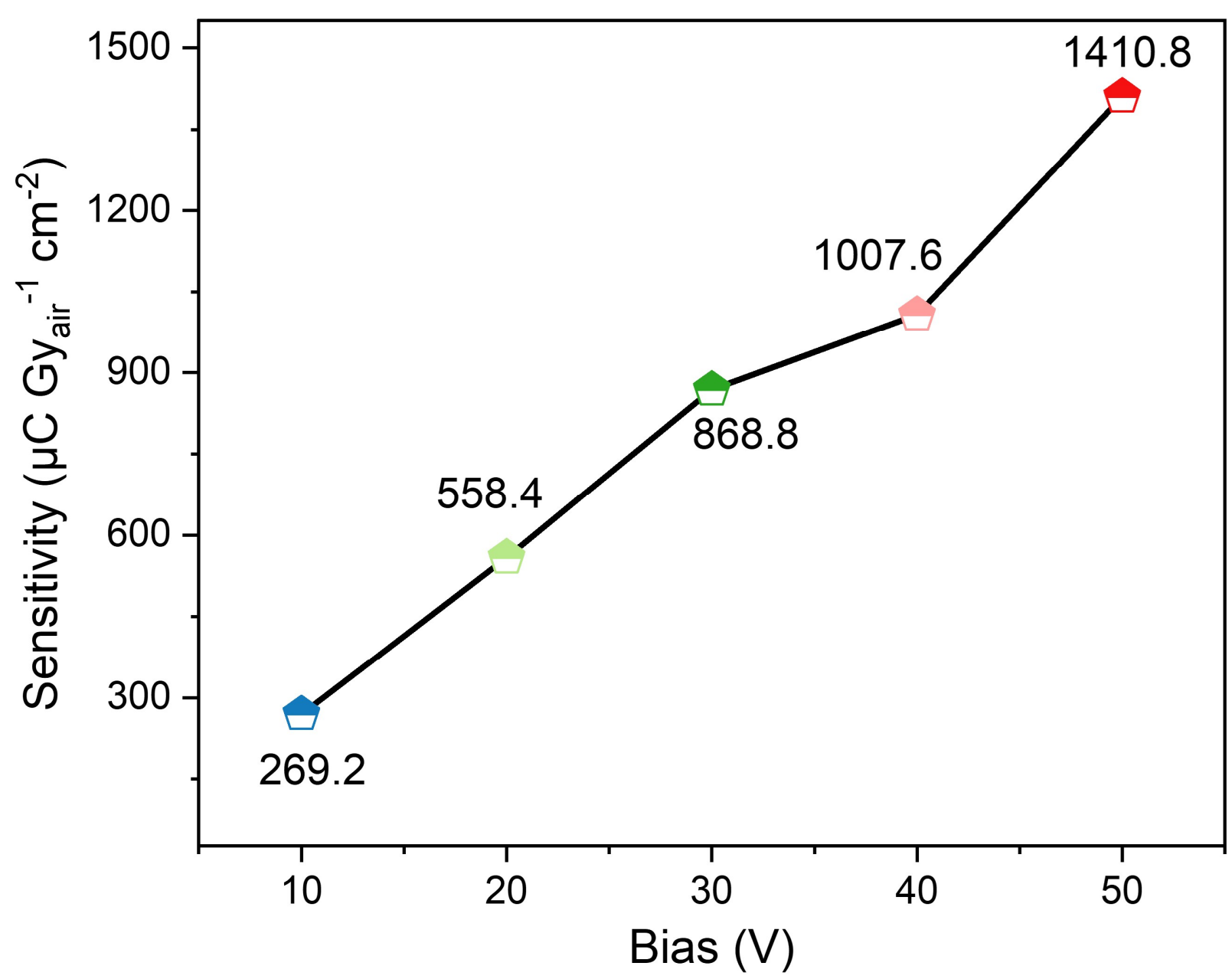

Figure S30. Correlation between the sensitivity and the applied bias voltage, showing a quasi-linear increase trend.

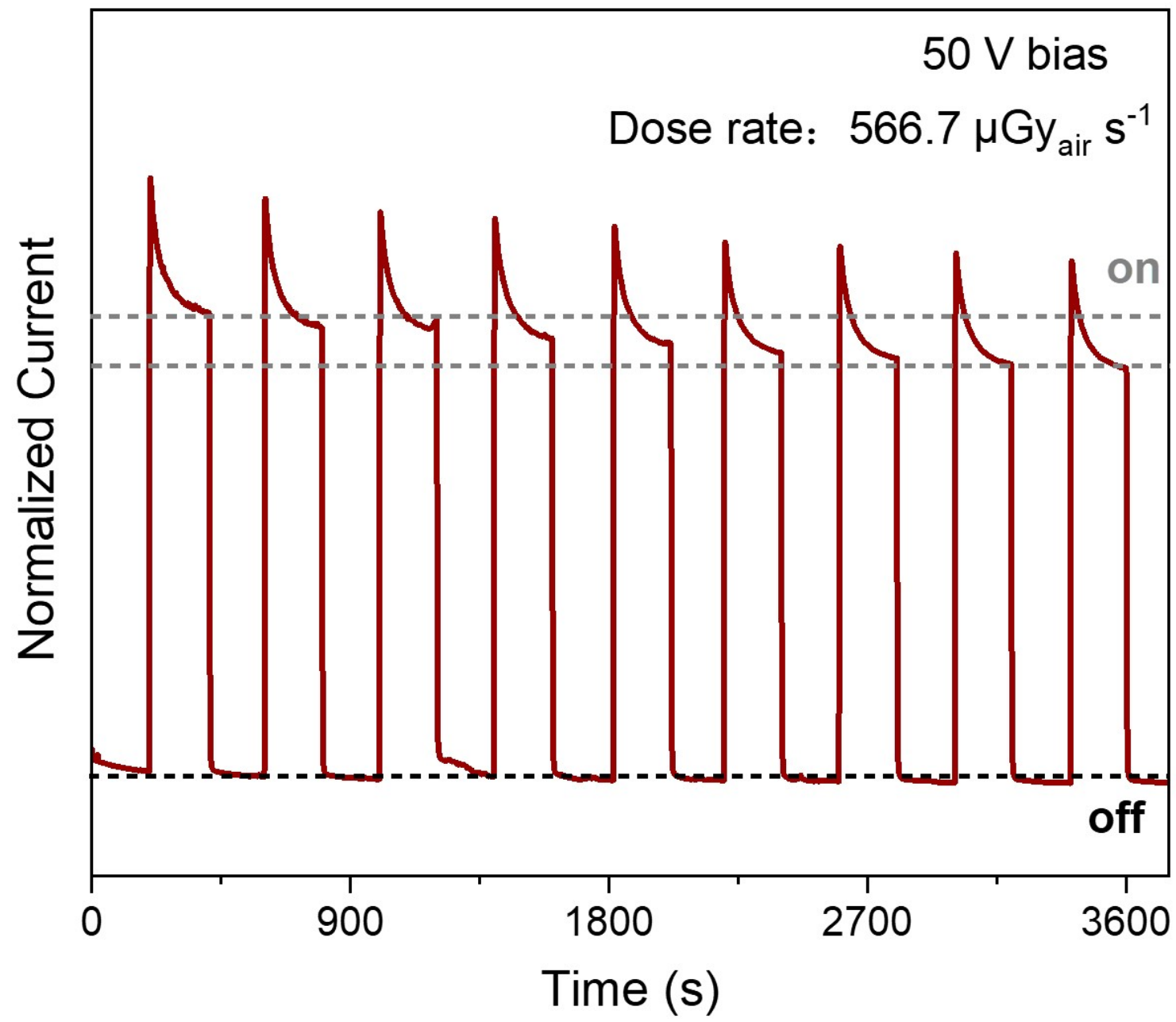

Figure S31. Operational stability of the (R-MBA)$_2$PtI$_6$ X-ray detector under continuous X-ray irradiation (566.7 μGyairs^{-1}, 100 V bias).

Table S1. Crystal data and structure refinement parameters for (*R*/*S*-MBA)$_2$PtI$_6$ and (*rac*-MBA)$_2$PtI$_6$.

Compound	(*R*-MBA)$_2$PtI$_6$	(*S*-MBA)$_2$PtI$_6$	(*rac*-MBA)$_2$PtI$_6$
**Formula**	$C_{16}H_{24}N_2PtI_6$	$C_{16}H_{24}N_2PtI_6$	$C_{16}H_{24}N_2PtI_6$
**Formula weight**	1200.86	1200.86	1200.86
**Temperature/K**	298.15	298.15	298.15
**Wavelength/Å**	0.71073	0.71073	0.71073
**Crystal system**	Trigonal	Trigonal	Trigonal
**Space group**	R3	R3	R-3
**a / Å**	13.8511(4)	13.8414(10)	13.751(4)
**b / Å**	13.8511(4)	13.8414(10)	13.751(4)
**c / Å**	36.7582(17)	36.744(4)	36.670(14)
**α / °**	90.0000	90.0000	90.0000
**β / °**	90.0000	90.0000	90.0000
**γ / °**	120.0000	120.0000	120.0000
**V / Å3**	6107.4(5)	6096.5(11)	6005(3)
**Dcalcg/cm^3**	2.938	2.944	2.989
**Z**	9	9	9
**F(000)**	4770.0	4770.0	4739.7
**Crystal size/mm^3**	0.13 × 0.12 × 0.1	0.13 × 0.12 × 0.1	0.45 × 0.32 × 0.27
**Radiation**	MoKα (λ = 0.71073)	MoKα (λ = 0.71073)	MoKα (λ = 0.71073)
**2Θ range for data collection/°**	5.6 to 55.1	5.6 to 55.1	5.6 to 50.4
**Index ranges**	-17 ≤ h ≤ 17	-18 ≤ h ≤ 17	-15 ≤ h ≤ 14
	-17 ≤ k ≤ 17	-17 ≤ k ≤ 18	-16 ≤ k ≤ 16
	-47 ≤ l ≤ 47	-47 ≤ l ≤ 47	-43 ≤ l ≤ 43
**Reflections collected**	87289	130583	13439
**Absorption coefficient**	11.998mm^{-1}	12.019mm^{-1}	12.132 mm^{-1}
**Independent reflections**	6214 [R_{int} = 0.0932, R_{sigma} = 0.0363]	6230 [R_{int} = 0.0907, R_{sigma} = 0.0249]	2395 [Rint = 0.0724, Rsigma = 0.0469]
**Data completeness**	1.99/1.00	2.00/1.00	0.982
**Theta(max)**	27.504	27.525	27.931
**Final R indexes [I>=2σ (I)]**	R_1 = 0.0262<br>wR_2 = 0.0515	R_1 = 0.0376<br>wR_2 = 0.0785	R1 = 0.0718<br>wR2 = 0.1666
**Final R indexes [all data]**	R_1=0.0262<br>wR_2=0.0554	R_1=0.0376<br>wR_2=0.0866	R1 = 0.0807<br>wR2 = 0.1710
**Completeness to Theta**	99.9%	99.9%	99.8%
**Goodness-of-fit on F^2**	1.026	1.153	1.088
**Flack parameter**	0.056(16)	0.08(3)	N/A

Table S2. Selected bond lengths for $(R\text{-MBA})_2PtI_6$.

Atom	Atom	Length/Å
Pt1	$I1^{1}$	2.6643(16)
Pt1	$I1^{2}$	2.6643(16)
Pt1	I1	2.6643(16)
Pt1	$I2^{2}$	2.6888(16)
Pt1	$I2^{1}$	2.6888(16)
Pt1	I2	2.6888(16)
Pt2	$I3^{1}$	2.6865(16)
Pt2	I3	2.6866(16)
Pt2	$I3^{2}$	2.6865(16)
Pt2	$I4^{1}$	2.6812(16)
Pt2	$I4^{2}$	2.6811(16)
Pt2	I4	2.6810(16)
Pt3	$I5^{3}$	2.6616(16)
Pt3	$I5^{4}$	2.6616(16)
Pt3	I5	2.6615(16)
Pt3	$I6^{4}$	2.6900(15)
Pt3	$I6^{3}$	2.6900(15)
Pt3	I6	2.6901(15)

Table S3. Selected bond angles for (R-MBA)$_2$PtI$_6$.

Atom	Atom	Atom	Angle/°
I1^{1}	Pt1	I1^{2}	90.37(6)
I1^{1}	Pt1	I1	90.37(6)
I1^{2}	Pt1	I1	90.37(6)
I1^{1}	Pt1	I2	88.46(5)
I1^{2}	Pt1	I2	178.11(5)
I1	Pt1	I2^{2}	88.46(5)
I1	Pt1	I2^{1}	178.11(5)
I1^{1}	Pt1	I2^{1}	91.13(5)
I1^{1}	Pt1	I2^{2}	178.11(5)
I1	Pt1	I2	91.12(5)
I1^{2}	Pt1	I2^{1}	88.46(5)
I1^{2}	Pt1	I2^{2}	91.13(5)
I2^{1}	Pt1	I2^{2}	90.07(6)
I2^{1}	Pt1	I2	90.07(6)
I2	Pt1	I2^{2}	90.07(6)
I3^{1}	Pt2	I3^{2}	91.72(6)
I3^{2}	Pt2	I3	91.72(6)
I3^{1}	Pt2	I3	91.72(6)
I4	Pt2	I3^{1}	88.53(4)
I4^{2}	Pt2	I3^{1}	88.28(4)
I4^{2}	Pt2	I3^{2}	179.75(8)
I4	Pt2	I3^{2}	88.28(4)
I4	Pt2	I3	179.75(8)
I4^{2}	Pt2	I3	88.53(4)
I4^{1}	Pt2	I3^{2}	88.53(4)
I4^{1}	Pt2	I3	88.28(4)
I4^{1}	Pt2	I3^{1}	179.75(8)
I4^{2}	Pt2	I4^{1}	91.46(6)
I4	Pt2	I4^{2}	91.47(6)
I4	Pt2	I4^{1}	91.47(6)
I5^{3}	Pt3	I5^{4}	90.28(6)
I5^{3}	Pt3	I5	90.28(6)
I5^{4}	Pt3	I5	90.28(6)
I5^{3}	Pt3	I6^{3}	89.11(5)
I5	Pt3	I6^{3}	89.90(5)

I5	Pt3	I6	89.11(5)
I5	Pt3	I6[4]	179.36(6)
I5[3]	Pt3	I6[4]	89.91(5)
I5[3]	Pt3	I6	179.36(5)
I5[4]	Pt3	I6[3]	179.36(5)
I5[4]	Pt3	I6[4]	89.11(5)
I5[4]	Pt3	I6	89.91(5)
I6[4]	Pt3	I6	90.70(5)
I6[4]	Pt3	I6[3]	90.71(5)
I6[3]	Pt3	I6	90.70(5)

Table S4. Selected bond lengths for (S-MBA)$_2$PtI$_6$.

Atom	Atom	Length/Å
Pt1	I1^{1}	2.679(2)
Pt1	I1^{2}	2.679(2)
Pt1	I1	2.679(2)
Pt1	I2^{2}	2.685(2)
Pt1	I2^{1}	2.685(2)
Pt1	I2	2.685(2)
Pt2	I3^{3}	2.663(2)
Pt2	I3	2.663(2)
Pt2	I3^{4}	2.663(2)
Pt2	I4^{3}	2.688(2)
Pt2	I4^{4}	2.688(2)
Pt2	I4	2.688(2)
Pt3	I5	2.686(2)
Pt3	I5^{5}	2.686(2)
Pt3	I5^{6}	2.686(2)
Pt3	I6	2.662(3)
Pt3	I6^{5}	2.662(3)
Pt3	I6^{6}	2.662(3)

Table S5. Selected bond angles for (*S*-MBA)$_2$PtI$_6$.

Atom	Atom	Atom	Angle/°
I1^{1}	Pt1	I1^{2}	91.56(8)
I1^{1}	Pt1	I1	91.56(8)
I1^{2}	Pt1	I1	91.56(8)
I1^{1}	Pt1	I2^{1}	88.47(6)
I1^{1}	Pt1	I2	88.36(6)
I1^{2}	Pt1	I2^{1}	88.36(6)
I1^{2}	Pt1	I2^{2}	88.47(6)
I1^{2}	Pt1	I2	179.92(11)
I1	Pt1	I2^{1}	179.92(12)
I1	Pt1	I2^{2}	88.36(6)
I1^{1}	Pt1	I2^{2}	179.92(11)
I1	Pt1	I2	88.47(6)
I2^{1}	Pt1	I2^{2}	91.61(8)
I2^{1}	Pt1	I2	91.61(8)
I2	Pt1	I2^{2}	91.61(8)
I3^{3}	Pt2	I3^{4}	90.31(8)
I3^{3}	Pt2	I3	90.31(8)
I3^{4}	Pt2	I3	90.31(8)
I3^{3}	Pt2	I4^{4}	90.07(7)
I3^{3}	Pt2	I4^{3}	89.04(7)
I3^{4}	Pt2	I4^{4}	89.04(7)
I3^{4}	Pt2	I4	90.07(7)
I3^{4}	Pt2	I4^{3}	179.25(8)
I3	Pt2	I4^{4}	179.25(8)
I3	Pt2	I4	89.04(7)
I3^{3}	Pt2	I4	179.24(8)
I3	Pt2	I4^{3}	90.07(7)
I4^{3}	Pt2	I4	90.58(7)
I4^{4}	Pt2	I4	90.58(7)
I4^{4}	Pt2	I4^{3}	90.58(7)
I5^{5}	Pt3	I5	90.23(9)
I5	Pt3	I5^{6}	90.23(8)
I5^{5}	Pt3	I5^{6}	90.23(9)
I6	Pt3	I5	88.54(8)
I6^{5}	Pt3	I5^{6}	90.94(8)
I6	Pt3	I5^{6}	178.31(9)
I6	Pt3	I5^{5}	90.94(8)

I6^{5}	Pt3	I5^{5}	88.54(8)
I6^{5}	Pt3	I5	178.31(9)
I6^{6}	Pt3	I5	90.93(8)
I6^{6}	Pt3	I5^{6}	88.54(8)
I6^{6}	Pt3	I5^{5}	178.31(9)
I6^{5}	Pt3	I6	90.32(10)
I6^{6}	Pt3	I6	90.32(10)
I6^{5}	Pt3	I6^{6}	90.32(10)

Table S6. Selected bond lengths for (*rac*-MBA)$_2$PtI$_6$.

Atom	Atom	Length/Å
Pt1	I1	2.6707(17)
Pt1	I1[1]	2.6707(17)
Pt1	I1[2]	2.6707(17)
Pt1	I2[1]	2.6509(18)
Pt1	I2	2.6509(18)
Pt1	I2[2]	2.6509(18)
Pt2	I3[3]	2.666(3)
Pt2	I3	2.666(3)
Pt2	I3[4]	2.666(3)
Pt2	I3[5]	2.666(3)
Pt2	I3[6]	2.666(3)
Pt2	I3[7]	2.666(3)

Table S7. Selected bond angles for (*rac*-MBA)$_2$PtI$_6$.

**Atom**	**Atom**	**Atom**	**Angle/°**
$I1^{1}$	Pt1	I1	90.29(6)
$I1^{2}$	Pt1	I1	90.29(6)
$I1^{2}$	Pt1	$I1^{1}$	90.29(6)
$I2^{1}$	Pt1	I1	88.77(5)
I2	Pt1	I1	178.65(5)
$I2^{1}$	Pt1	$I1^{1}$	178.65(5)
$I2^{2}$	Pt1	$I1^{1}$	88.77(5)
I2	Pt1	$I1^{1}$	90.68(5)
$I2^{1}$	Pt1	$I1^{2}$	90.68(5)
$I2^{2}$	Pt1	$I1^{2}$	178.65(5)
$I2^{2}$	Pt1	I1	90.68(5)
I2	Pt1	$I1^{2}$	88.77(5)
$I2^{2}$	Pt1	$I2^{1}$	90.27(6)
I2	Pt1	$I2^{1}$	90.27(7)
$I2^{2}$	Pt1	I2	90.27(7)
I3	Pt2	$I3^{3}$	88.59(10)
$I3^{4}$	Pt2	$I3^{5}$	88.59(10)
$I3^{6}$	Pt2	$I3^{4}$	91.41(10)
$I3^{4}$	Pt2	$I3^{7}$	88.59(10)
$I3^{6}$	Pt2	I3	88.59(10)
$I3^{6}$	Pt2	$I3^{7}$	88.59(10)
$I3^{5}$	Pt2	$I3^{3}$	88.59(10)
$I3^{7}$	Pt2	$I3^{3}$	180.0
$I3^{5}$	Pt2	I3	91.41(10)
$I3^{6}$	Pt2	$I3^{3}$	91.41(10)
$I3^{5}$	Pt2	$I3^{7}$	91.41(10)
$I3^{7}$	Pt2	I3	91.41(10)
$I3^{6}$	Pt2	$I3^{5}$	180.0
$I3^{4}$	Pt2	I3	180.0
$I3^{4}$	Pt2	$I3^{3}$	91.41(10)

Table S8. The distortion parameters of [PtI_6] octahedra for (R/S-MBA)$_2$$PtI_6$ and (rac-MBA)$_2$$PtI_6$.

Compound	Octahedra	Proportion	λ_{oct} value ($\times10^{-5}$)	σ^2 value
(R-MBA)$_2$$PtI_6$	[Pt(1)I_6]	1/3	3.48	1.03
	[Pt(2)I_6]	1/3	3.48	0.59
	[Pt(3)I_6]	1/3	0.69	2.79
(S-MBA)$_2$$PtI_6$	[Pt(1)I_6]	1/3	3.48	0.86
	[Pt(2)I_6]	1/3	3.48	0.37
	[Pt(3)I_6]	1/3	0.69	2.74
(rac-MBA)$_2$$PtI_6$	[Pt(1)I_6]	2/3	1.41	0.58
	[Pt(2)I_6]	1/3	0	1.27

Table S9. Comparison of the bandgap values of (R/S-MBA)$_2$PtI$_6$ with the reported perovskite halides.

Structure type	Compound	Bandgap type	Bandgap (eV)	Ref.
3D	$FAPbI_3$	Direct	1.48	10, 11
	$FASnI_3$	Direct	1.41	12, 13
	$MPSnI_3$	Direct	1.43	14
	$Cs_2AgTlBr_6$	Direct	0.95	15
	Cs_2TlBiI_6	Direct	1.10	16
	$Cs_2AgSbBr_6$	Indirect	1.64	17
	$Cs_2CuSbCl_6$	Indirect	1.66	18
2D	(4,4-difluoropiperidinium)$_4$AgBiI$_8$	Indirect	2.03	19
	(BA)$_2$(EA)$_2$Pb$_3$I$_{10}$	/	1.9	20
	[n-pentylaminium]$_2$[ethylammonium]$_2$Pb$_3$I$_{10}$	Direct	1.86	21
	HA$_2$EA$_2$Pb$_3$I$_{10}$	Direct	1.83	22
	(isopentylammonium)$_2$(ethylammonium)$_2$Pb$_3$I$_{10}$	Direct	1.80	23
	(BA)$_2$(FA)$_2$Pb3I$_{10}$	/	1.51	24
1D	FcPbI$_3$	/	1.48	25
	[R/S-BEPA]$_2$FAPb$_2$I$_7$	Direct	1.45	26
	(HDA)SbI$_5$	Indirect	1.81	27
	(HDA)SbI$_5$	Indirect	1.66	27
	(4-AMP)BiI$_5$	/	1.81	28
0D	[N,N-dimethylimidazole]$_3$Bi$_2$I$_9$	/	2.1	29
	$Cs_3Bi_2I_9$	Direct	2.1	30
	Cs_2RuCl_6	Direct	1.43	31
	Cs_2PtI_6	Direct	1.41	32
	(R/S-MBA)$_2$PtI$_6$	/	1.27	This work

Table S10. Comparison of g_{lph} values for CPL detection based on (R-MBA)$_2$PtI$_6$ and the reported chiral hybrid perovskite halides.

Structure type	Compound	λ (nm)	g_{lph}	Ref.
2D	(R-PPA)EAPbBr$_4$	266	0.42	33
	(R-MPA)$_4$AgBiI$_8$	520	0.3	34
	S-[(4-aminophenyl)ethylamine]$_2$AgBiI$_8$·0.5H$_2$O	520	0.27	35
	(R-MPA)$_2$MAPb$_2$I$_7$	532	0.2	36
	(R)-3BrMBA$_2$PbBr$_4$	395	0.18	37
	R-(BrBA)$_2$PbBr$_4$	405	0.14	38
	(R-BPEA)$_2$PbI$_4$	520	0.13	39
	[(R)-β-MPA]$_2$PbCl$_4$	375	0.1	40
1D	(R-NEA)PbI$_3$	405	0.29	41
	(R-C$_5$H$_{14}$N)PbI$_3$	405	0.23	42
	(R/S-BEPA$_2$FAPb$_2$I$_7$	785	0.21	26
	(S-PPA)$_4$(IPA)$_6$Ag$_2$Bi$_4$I$_{24}$·2H$_2$O	520	0.21	43
	(R-PEA)PbI$_3$	395	0.1	44
	(R-MBA)BiBr$_4$	380	0.07	11
0D	(R-MBA)$_4$Bi$_2$Br$_{10}$	380	0.25	11
	(R-MBA)$_2$CuCl$_4$	405	0.21	45
	(R-α-MBA)$_2$PtI$_6$	520	0.26	This work

References

(1) Ji, D.; Li, T.; Liu, J.; Amirjalayer, S.; Zhong, M.; Zhang, Z. Y.; Huang, X.; Wei, Z.; Dong, H.; Hu, W.; Fuchs, H., Band-like Transport in Small-Molecule Thin Films toward High Mobility and Ultrahigh Detectivity Phototransistor Arrays. *Nat. Commun.* **2019**, *10*, 12.

(2) Pan, W.; Wu, H.; Luo, J.; Deng, Z.; Ge, C.; Chen, C.; Jiang, X.; Yin, W.-J.; Niu, G.; Zhu, L., $Cs_2AgBiBr_6$ Single-Crystal X-Ray Detectors with A Low Detection Limit. *Nat. Photonics* **2017**, *11*, 726–732.

(3) Yakunin, S.; Sytnyk, M.; Kriegner, D.; Shrestha, S.; Richter, M.; Matt, G. J.; Azimi, H.; Brabec, C. J.; Stangl, J.; Kovalenko, M. V.; Heiss, W., Detection of X-Ray Photons by Solution-Processed Organic-Inorganic Perovskites. *Nat. Photonics* **2015**, *9*, 444–449.

(4) Kresse, G.; Furthmuller, J., Efficient Iterative Schemes for ab Initio Total-Energy Calculations using A Plane-Wave Basis Set. *Phys. Rev. B Condens. Matter* **1996**, *54*, 11169–11186.

(5) Kresse, G.; Joubert, D., From Ultrasoft Pseudopotentials to The Projector Augmented-Wave Method. *Phys. Rev. B* **1999**, *59*, 1758.

(6) Kresse, G.; Furthmüller, J., Efficiency of ab-Initio Total Energy Calculations for Metals and Semiconductors using A Plane-Wave Basis Set. *Comput. Mater. Sci.* **1996**, *6*, 15–50.

(7) Perdew, J. P.; Burke, K.; Ernzerhof, M., Generalized Gradient Approximation Made Simple. *Phys. Rev. Lett.* **1996**, 77 (18), 3865.

(8) Grimme, S., Semiempirical GGA-Type Density Functional Constructed with A Long-Range Dispersion Correction. *J. Comput. Chem.* **2006**, *27*, 1787–99.

(9) Krukau, A. V.; Vydrov, O. A.; Izmaylov, A. F.; Scuseria, G. E., Influence of The Exchange Screening Parameter on The Performance of Screened Hybrid Functionals. *J. Chem. Phys.* **2006**, *125*, 224106.

(10) Fang, H. H.; Wang, F.; Adjokatse, S.; Zhao, N.; Even, J.; Antonietta Loi, M., Photoexcitation Dynamics in Solution-Processed Formamidinium Lead Iodide Perovskite Thin Films for Solar Cell Applications. *Light: Sci. Appl.* **2016**, *5*, e16056.

(11) Maiti, A.; Pal, A. J., Spin-Selective Charge Transport in Lead-Free Chiral Perovskites: The Key towards High-Anisotropy in Circularly-Polarized Light Detection. *Angew. Chem., Int. Ed.* **2022**, *61*, e202214161.

(12) Ke, W.; Stoumpos, C. C.; Zhu, M.; Mao, L.; Spanopoulos, I.; Liu, J.; Kontsevoi, O. Y.; Chen, M.; Sarma, D.; Zhang, Y.; Wasielewski, M. R.; Kanatzidis, M. G., Enhanced Photovoltaic Performance and Stability with A New Type of Hollow 3D Perovskite {en}$FASnI_3$. *Sci. Adv.* **2017**, *3*, e1701293.

(13) Wu, T.; Liu, X.; Luo, X.; Segawa, H.; Tong, G.; Zhang, Y.; Ono, L. K.; Qi, Y.; Han, L., Heterogeneous $FASnI_3$ Absorber with Enhanced Electric Field for High-Performance Lead-Free Perovskite Solar Cells. *Nano-Micro Lett.* **2022**, *14*, 99.

(14) Zhang, H. Y.; Xiong, R. G., Three-Dimensional Narrow-Bandgap Perovskite Semiconductor Ferroelectric Methylphosphonium Tin Triiodide for Potential Photovoltaic Application. *Chem. Commun.* **2023**, *59*, 920–923.

(15) Slavney, A. H.; Leppert, L.; Saldivar Valdes, A.; Bartesaghi, D.; Savenije, T. J.; Neaton, J. B.; Karunadasa, H. I., Small-Band-Gap Halide Double Perovskites. *Angew. Chem., Int. Ed.* **2018**, *57*, 12765–12770.

(16) Reynolds, R. P.; Persaud, J. H.; Volonakis, G.; Torma, A. J.; Meza, P. E.; Kelly, Z. E.; Stoumpos, C. C.; Dravid, V. P.; Katan, C.; Mohite, A. D.; Kanatzidis, M. G., Design Principles for Narrow-Gap Hybrid Semiconductors: Insights from Viologen-Tin and Viologen-Lead Iodides. *J. Am. Chem. Soc.* **2025**, *147*, 24573–24593.

(17) Wei, F.; Deng, Z.; Sun, S.; Hartono, N. T. P.; Seng, H. L.; Buonassisi, T.; Bristowe, P. D.; Cheetham, A. K., Enhanced Visible Light Absorption for Lead-Free Double Perovskite $Cs_2AgSbBr_6$. *Chem. Commun.* **2019**, *55*, 3721–3724.

(18) Zhou, W.; Han, P.; Zhang, X.; Zheng, D.; Yang, S.; Yang, Y.; Luo, C.; Yang, B.; Hong, F.; Wei, D.; Lu, R.; Han, K., Lead-Free Small-Bandgap $Cs_2CuSbCl_6$ Double Perovskite Nanocrystals. *J. Phys. Chem. Lett.* **2020**, *11*, 6463–6467.

(19) Wang, C. F.; Li, H.; Li, M. G.; Cui, Y.; Song, X.; Wang, Q. W.; Jiang, J. Y.; Hua, M. M.; Xu, Q.; Zhao, K.; Ye, H. Y.; Zhang, Y., Centimeter-Sized Single Crystals of Two-Dimensional Hybrid Iodide Double Perovskite (4,4-Difluoropiperidinium)$_4AgBiI_8$ for High-Temperature Ferroelectricity and Efficient X-Ray Detection. *Adv. Funct. Mater.* **2021**, *31*, 2009457.

(20) Han, S.; Liu, X.; Liu, Y.; Xu, Z.; Li, Y.; Hong, M.; Luo, J.; Sun, Z., High-Temperature Antiferroelectric of Lead Iodide Hybrid

Perovskites. *J. Am. Chem. Soc.* **2019**, *141*, 12470−12474.

(21) Han, S.; Li, L.; Ji, C.; Liu, X.; Wang, G. E.; Xu, G.; Sun, Z.; Luo, J., Visible-Photoactive Perovskite Ferroelectric-Driven Self-Powered Gas Detection. *J. Am. Chem. Soc.* **2023**, *145*, 12853−12860.

(22) Han, S.; Ma, Y.; Hua, L.; Tang, L.; Wang, B.; Sun, Z.; Luo, J., Soft Multiaxial Molecular Ferroelectric Thin Films with Self-Powered Broadband Photodetection. *J. Am. Chem. Soc.* **2022**, *144*, 20315−20322.

(23) Han, S.; Li, M.; Liu, Y.; Guo, W.; Hong, M. C.; Sun, Z.; Luo, J., Tailoring of A Visible-Light-Absorbing Biaxial Ferroelectric towards Broadband Self-Driven Photodetection. *Nat. Commun.* **2021**, *12*, 284.

(24) Yan, J.; Fu, W.; Zhang, X.; Chen, J.; Yang, W.; Qiu, W.; Wu, G.; Liu, F.; Heremans, P.; Chen, H., Highly Oriented Two-Dimensional Formamidinium Lead Iodide Perovskites with A Small Bandgap of 1.51 eV. *Mater. Chem. Front.* **2018**, *2*, 121−128.

(25) Xu, Z. K.; Wang, Z. X., A Low-Dimensional Ferrocenium Lead-Iodide Perovskite Ferroelastic with A Narrow Band Gap. *Chem. Commun.* **2025**, *61*, 15810−15813.

(26) Ye, H.; Peng, Y.; Wei, M.; Zhang, X.; Zhu, T.; Guan, Q.; Li, L.; Chen, S.; Liu, X.; Luo, J., Bulk Photovoltaic Effect in Chiral Layered Hybrid Perovskite Enables Highly Sensitive Near-Infrared Circular Polarization Photodetection. *Chem. Mater.* **2023**, *35*, 6591−6597.

(27) Wang, C. F.; Li, H.; Ning, C.; Wang, L.; Luo, Q. F.; Li, T.; Ni, H. F.; An, Z.; Zhang, Z. X.; Wang, J.; Fu, D. W.; Ju, M. G.; Zhao, K.; Zhang, Y., Eco-Friendly Preparation of High-Quality Lead-Free Halide Ferroelectric Solid-Solution Toward High-Performance X-ray Detection. *Angew. Chem., Int. Ed.* **2026**, *65*, e25694.

(28) Zhu, T.; Bie, J.; Ji, C.; Zhang, X.; Li, L.; Liu, X.; Huang, X. Y.; Fa, W.; Chen, S.; Luo, J., Circular Polarized Light-Dependent Anomalous Photovoltaic Effect from Achiral Hybrid Perovskites. *Nat. Commun.* **2022**, *13*, 7702.

(29) Yang, W.; Chu, K.-B.; Zhang, L.; Ding, X.; Sun, J.; Liu, J. Z.; Song, J.-L.; Zheng, C.; Deng, J., Lead-Free Molecular Ferroelectric [N, N-Dimethylimidazole]$_3Bi_2I_9$ with Narrow Bandgap. *Mater. Des.* **2020**, *193*, 108868.

(30) McCall, K. M.; Stoumpos, C. C.; Kontsevoi, O. Y.; Alexander, G. C. B.; Wessels, B. W.; Kanatzidis, M. G., From 0D $Cs_3Bi_2I_9$ to 2D $Cs_3Bi_2I_6Cl_3$: Dimensional Expansion Induces a Direct Band Gap but Enhances Electron–Phonon Coupling. *Chem. Mater.* **2019**, *31*, 2644−2650.

(31) Halpati, J. S.; Shanmugam, N.; Manoj, M.; Hamdan, M.; Chandiran, A. K., Mixed-Tetravalent $Cs_2Ru_mPt_{1-m}X_6$ ($X = Cl^-$, Br^-)-Based Vacancy-Ordered Halide Double Perovskites for Enhanced Solar Water Oxidation. *Sol. RRL* **2024**, *8*, 2300924.

(32) Liu, H.; Bansal, S., Pt and Pt-Group Transition Metal 0D Vacancy Ordered Halide Perovskites: A Review. *EcoMat* **2024**, *6*, e12492.

(33) Zhu, T.; Weng, W.; Ji, C.; Zhang, X.; Ye, H.; Yao, Y.; Li, X.; Li, J.; Lin, W.; Luo, J., Chain-to-Layer Dimensionality Engineering of Chiral Hybrid Perovskites to Realize Passive Highly Circular-Polarization-Sensitive Photodetection. *J. Am. Chem. Soc.* **2022**, *144*, 18062−18068.

(34) Li, D.; Liu, X.; Wu, W.; Peng, Y.; Zhao, S.; Li, L.; Hong, M.; Luo, J., Chiral Lead-Free Hybrid Perovskites for Self-Powered Circularly Polarized Light Detection. *Angew. Chem., Int. Ed.* **2021**, *60*, 8415−8418.

(35) Li, Z.; Ji, C.; Fan, Y.; Zhu, T.; You, S.; Wu, J.; Li, R.; Zhu, Z. K.; Yu, P.; Kuang, X.; Luo, J., Circularly Polarized Light-Dependent Pyro-Phototronic Effect from 2D Chiral-Polar Double Perovskites. *J. Am. Chem. Soc.* **2023**, *145*, 25134−25142.

(36) Wang, L.; Xue, Y.; Cui, M.; Huang, Y.; Xu, H.; Qin, C.; Yang, J.; Dai, H.; Yuan, M., A Chiral Reduced-Dimension Perovskite for an Efficient Flexible Circularly Polarized Light Photodetector. *Angew. Chem., Int. Ed.* **2020**, *59*, 6442−6450.

(37) Wu, J.; Chang, T.; Yang, Z.; Zan, H.; Bai, Y.; Han, X.; Luo, N.; Yuan, M.; Zeng, R., Magneto-Chiroptical Hybrid Perovskites with Anomalous Photovoltaic Effect for High-Performance Self-Driven Photodetectors. *Adv. Mater.* **2025**, *37*, e2509074.

(38) Gu, Q.; Chen, K.; Zhang, X.; Wang, S.; Wu, S.; Huang, W., Butylammonium-Based Chiral 2D Perovskite Single Crystals for Efficient UV Circularly Polarized Light Differentiation and High-Performance X-ray Detection. *ACS Appl. Mater. Interfaces* **2025**, *17*, 17127−17134.

(39) Peng, Y.; Liu, X.; Li, L.; Yao, Y.; Ye, H.; Shang, X.; Chen, X.; Luo, J., Realization of vis-NIR Dual-Modal Circularly Polarized Light Detection in Chiral Perovskite Bulk Crystals. *J. Am. Chem. Soc.* **2021**, *143*, 14077−14082.

(40) Zhang, X.; Weng, W.; Li, L.; Wu, H.; Yao, Y.; Wang, Z.; Liu, X.; Lin, W.; Luo, J., Heterogeneous Integration of Chiral Lead-Chloride Perovskite Crystals with Si Wafer for Boosted Circularly Polarized Light Detection in Solar-Blind Ultraviolet Region. *Small* **2021**, *17*,

e2102884.

(41) Li, M.; Fang, F.; Huang, X.; Liu, G.; Lai, Z.; Chen, Z.; Hong, J.; Chen, Y.; Wei, R.-j.; Ning, G.-H., Chiral Ligand-Induced Structural Transformation of Low-Dimensional Hybrid Perovskite for Circularly Polarized Photodetection. *Chem. Mater.* **2022**, *34*, 2955–2962.

(42) Xu, F.; Liu, H.; Jin, Z.; Huang, T.; Tang, C.; Tan, C. L.; Shi, Y.; Yan, S., Chiral Materials: Multidisciplinary Progress and Emerging Frontier Application Prospects. *Nanomaterials (Basel)* **2025**, *15*, 1701.

(43) Zhu, Z. K.; Zhu, T.; Wu, J.; You, S.; Yu, P.; Liu, X.; Li, L.; Ji, C.; Luo, J., Discovering New Type of Lead-Free Cluster-Based Hybrid Double Perovskite Derivatives with Chiral Optical Activities and Low X-Ray Detection Limit. *Adv. Funct. Mater.* **2023**, *33*, 2214660.

(44) Chen, C.; Gao, L.; Gao, W.; Ge, C.; Du, X.; Li, Z.; Yang, Y.; Niu, G.; Tang, J., Circularly Polarized Light Detection using Chiral Hybrid Perovskite. *Nat. Commun.* **2019**, *10*, 1927.

(45) Hao, J.; Lu, H.; Mao, L.; Chen, X.; Beard, M. C.; Blackburn, J. L., Direct Detection of Circularly Polarized Light Using Chiral Copper Chloride-Carbon Nanotube Heterostructures. *ACS Nano* **2021**, *15*, 7608–7617.
